# Supplementary material for: An Hsp90 co-chaperone protein in yeast is functionally replaced by site-specific posttranslational modification in humans
Source: Nat Commun. 2017 May 24;8:15328. doi: 10.1038/ncomms15328 (PMC5458067; doi:10.1038/ncomms15328)
Supplement: Supplementary Information — Supplementary figures, supplementary tables, supplementary methods and supplementary references. [file ncomms15328-s1.pdf]

Supplementary Information

Supplementary Figures

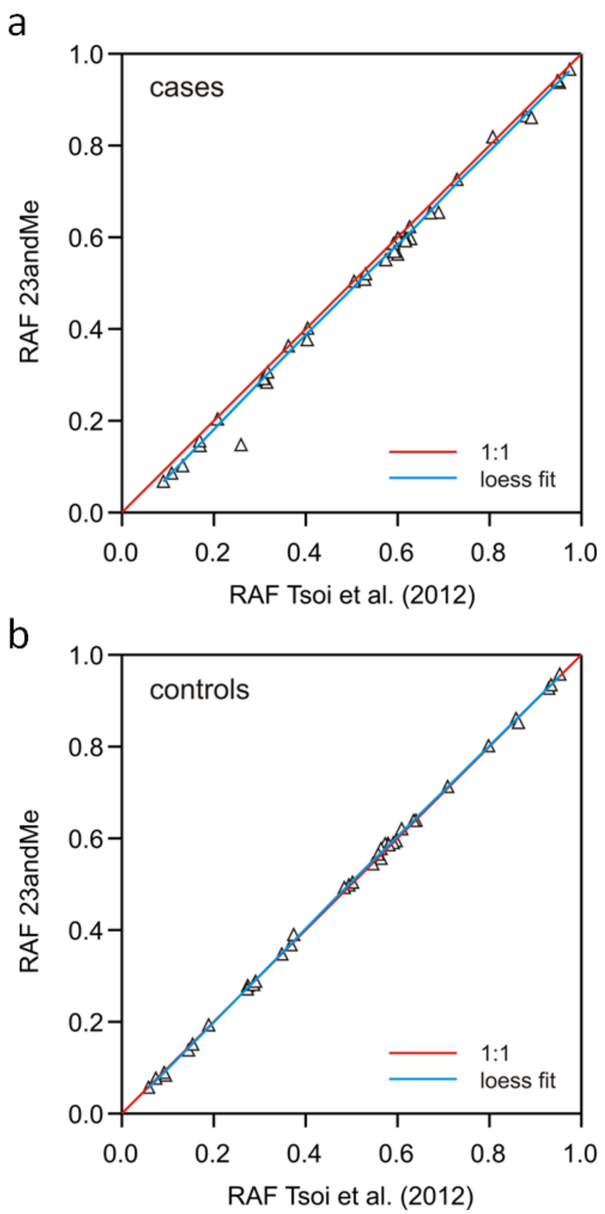

Supplementary Figure 1. Risk allele frequencies (RAFs) for the known psoriasis loci estimated in the 23andMe cohort versus those estimated from our previous study (Tsoi et al., 2012. *Nat Genet*) in cases (a) and controls (b). The loess fit is shown in blue, and the expected 1:1 relationship is shown in red.

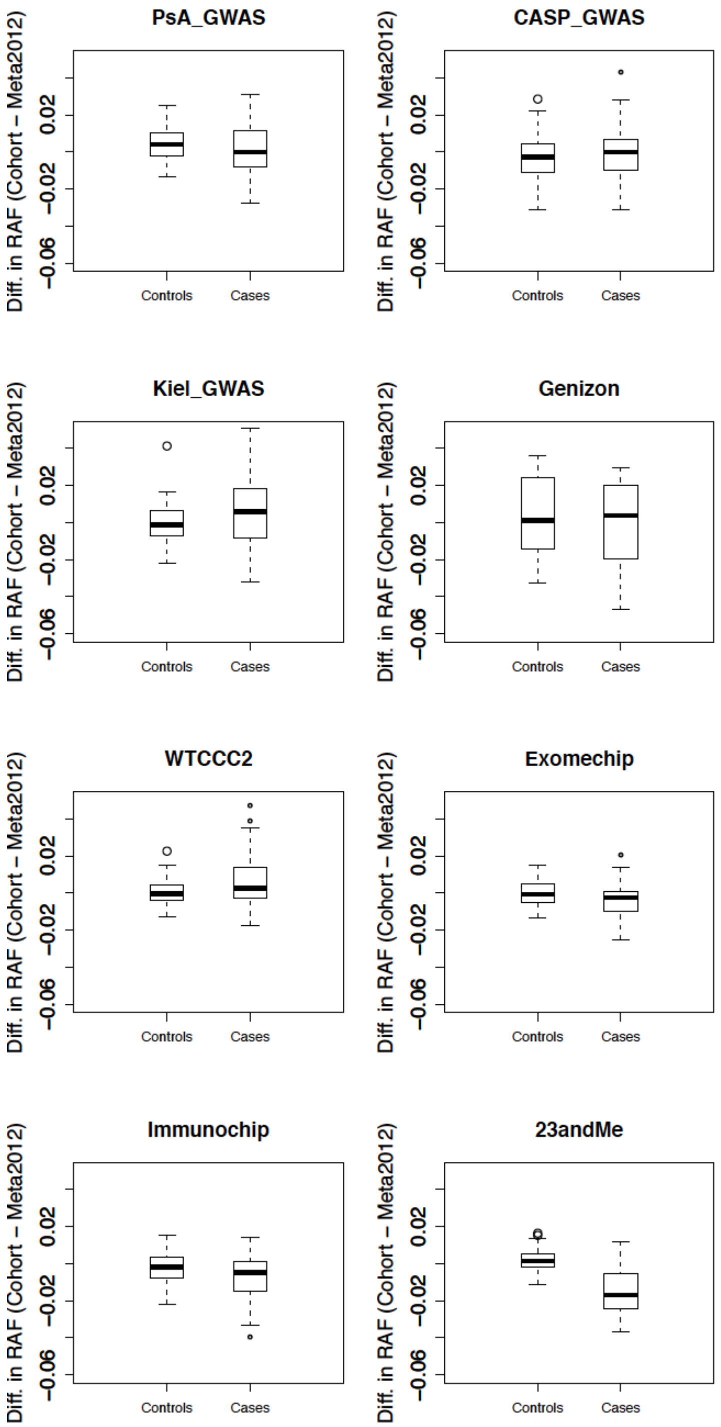

Supplementary Figure 2. Boxplots showing differences in risk allele frequencies (RAFs) estimated in the different cohorts versus those estimated in the meta-analysis of Tsoi et al, 2012. *Nat Genet*. The RAFs estimated from the meta-analysis were used as they represent the results of the previous largest genetic study of psoriasis in European-origin individuals.

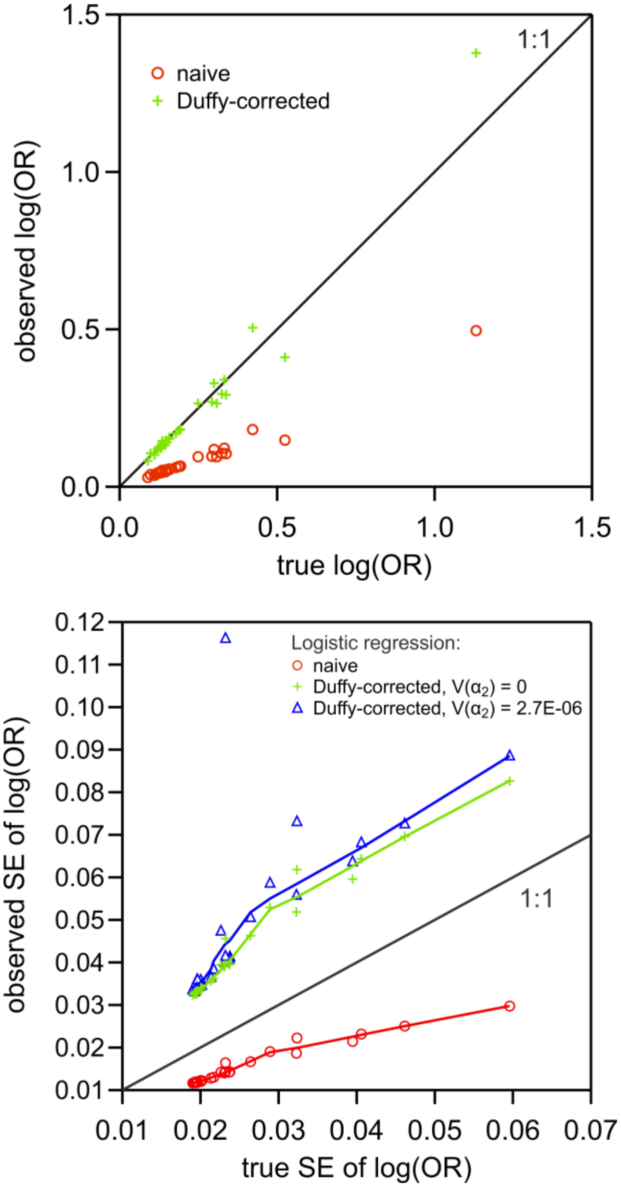

Supplementary Figure 3. Duffy-adjusted ORs and standard errors. (Top) Using ORs estimated from independent cohorts for known loci, the Duffy adjustment could correct the downward bias of ORs estimator in the 23andMe cohort. The results were based on simulation of misclassified data described in Materials and methods. (Bottom) Duffy-adjusted standard errors of the ln(OR), illustrating that correction for misclassification increases the standard error (blue and green vs. red), and the increase is greater when uncertainty for the estimator of the misclassification parameter α_2 is accounted for (blue vs. green).

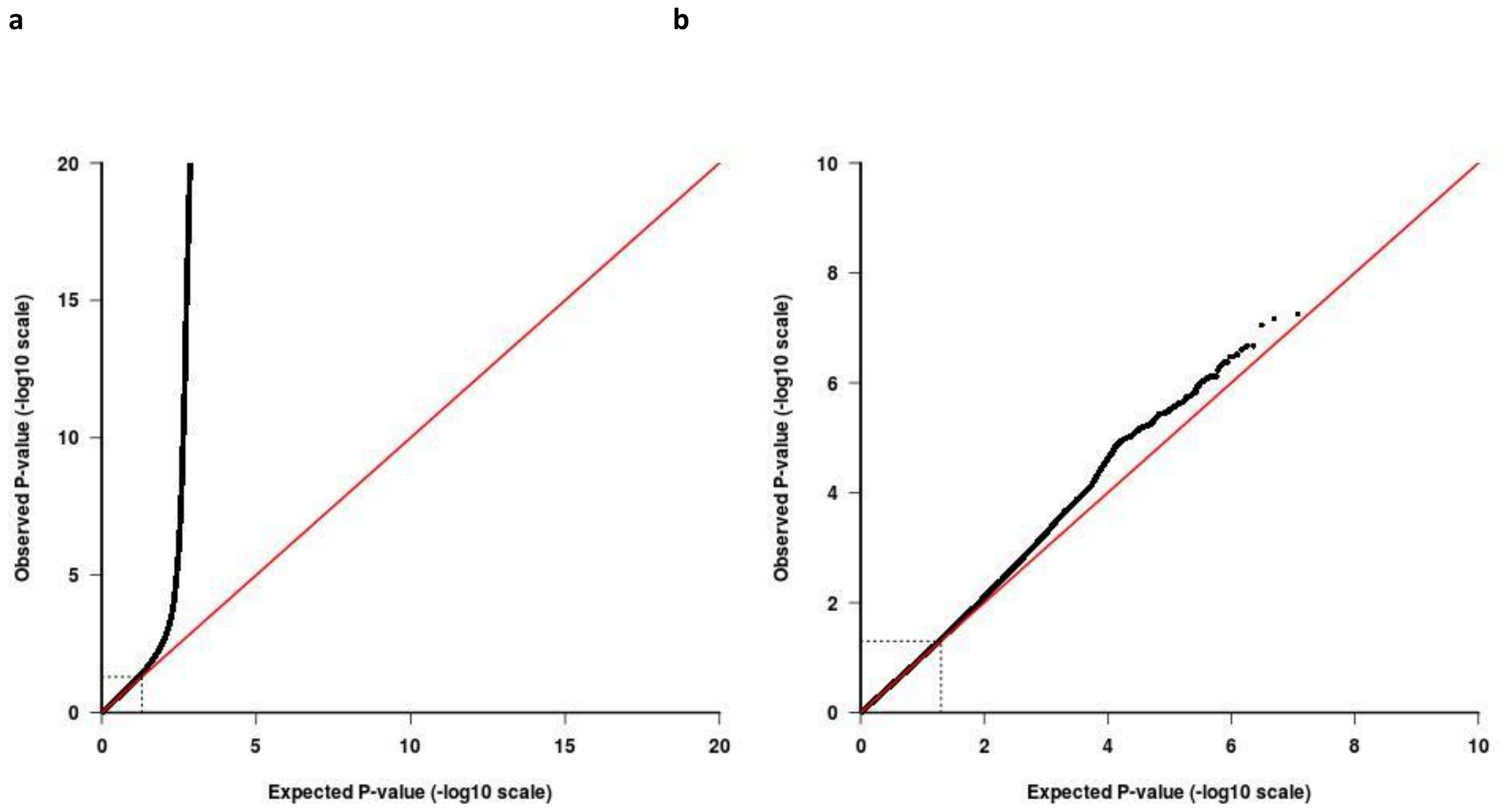

Supplementary Figure 4. QQ plots for the meta-analysis. a) all markers; b) markers outside previously known psoriasis susceptibility regions.

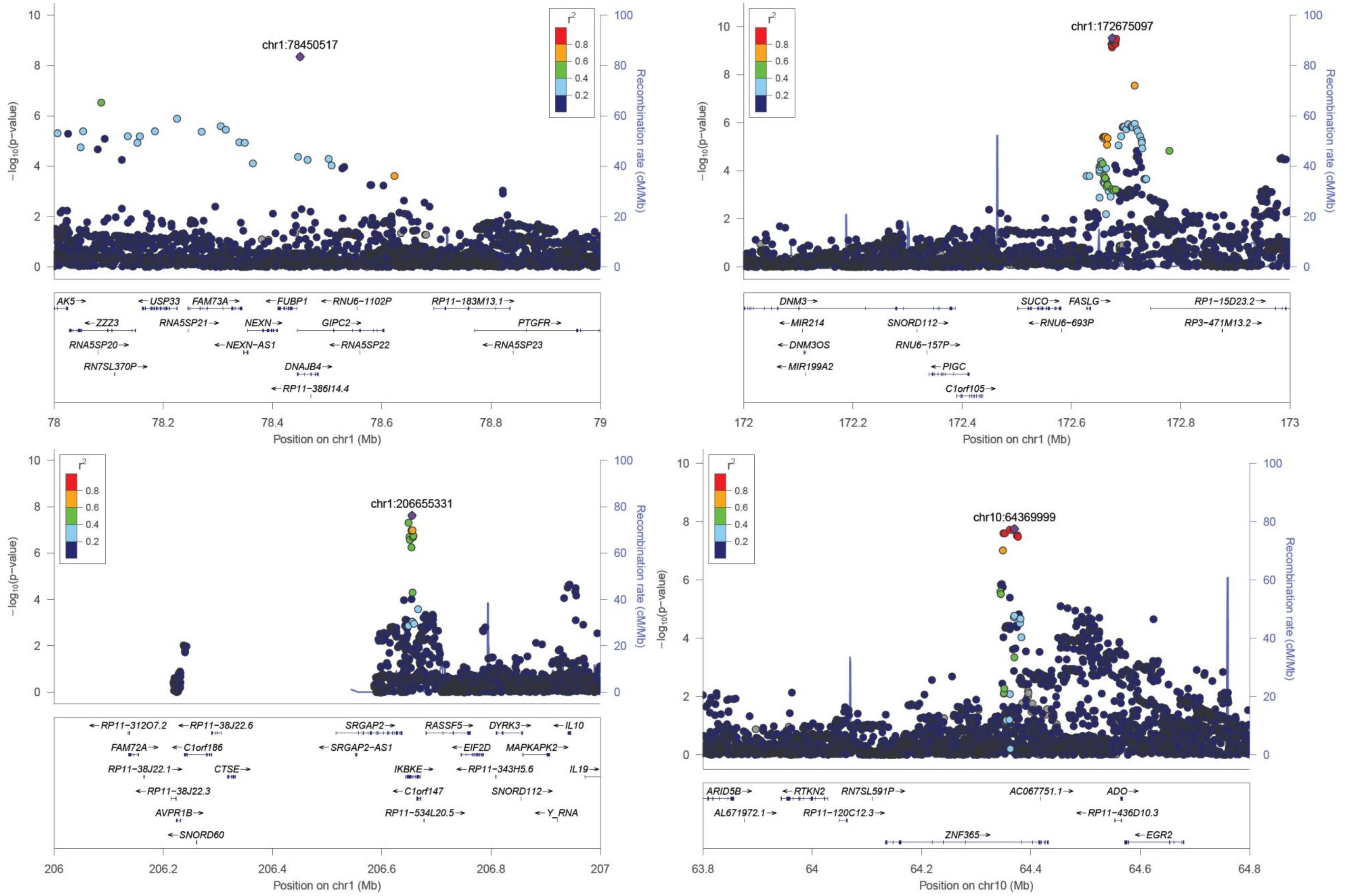

chr1:78450517
−log10(p−value)
Recombination rate (cM/Mb)
r2
0.8
0.6
0.4
0.2
AK5
USP33
FAM73A
FUBP1
RNU6−1102P
RP11−183M13.1
ZZZ3
RNA5SP21
NEXN
GIPC2
PTGFR
RNA5SP20
NEXN−AS1
RNA5SP22
RNA5SP23
RN7SL370P
DNAJB4
RP11−386I14.4
Position on chr1 (Mb)
chr1:172675097
DNM3
SUCO
FASLG
RP1−15D23.2
MIR214
SNORD112
RNU6−693P
RP3−471M13.2
DNM3OS
RNU6−157P
MIR199A2
PIGC
C1orf105
chr1:206655331
RP11−312O7.2
RP11−38J22.6
SRGAP2
RASSF5
DYRK3
IL10
FAM72A
C1orf186
SRGAP2−AS1
EIF2D
MAPKAPK2
RP11−38J22.1
CTSE
IKBKE
RP11−343H5.6
IL19
RP11−38J22.3
C1orf147
SNORD112
AVPR1B
RP11−534L20.5
Y_RNA
SNORD60
chr10:64369999
ARID5B
RTKN2
RN7SL591P
AC067751.1
ADO
AL671972.1
RP11−120C12.3
RP11−436D10.3
ZNF365
EGR2
Position on chr10 (Mb)

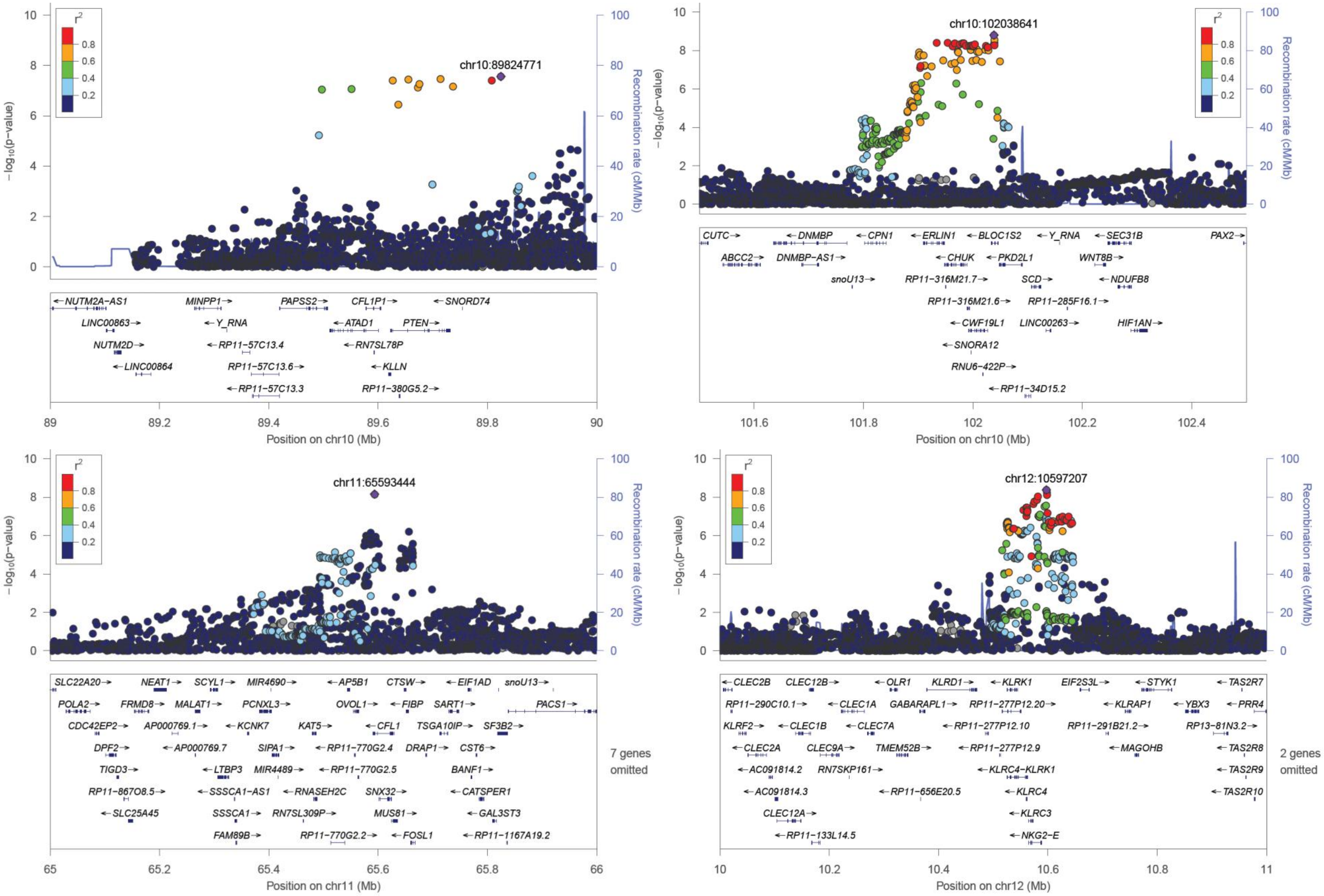

chr10:89824771
chr10:102038641
chr11:65593444
chr12:10597207
r2
0.8
0.6
0.4
0.2
−log10(p−value)
Recombination rate (cM/Mb)
Position on chr10 (Mb)
Position on chr10 (Mb)
Position on chr11 (Mb)
Position on chr12 (Mb)
NUTM2A−AS1
MINPP1
PAPSS2
CFL1P1
SNORD74
LINC00863
Y_RNA
ATAD1
PTEN
NUTM2D
RP11−57C13.4
RN7SL78P
LINC00864
RP11−57C13.6
KLLN
RP11−57C13.3
RP11−380G5.2
CUTC
DNMBP
CPN1
ERLIN1
BLOC1S2
Y_RNA
SEC31B
PAX2
ABCC2
DNMBP−AS1
CHUK
PKD2L1
WNT8B
snoU13
RP11−316M21.7
SCD
NDUFB8
RP11−316M21.6
RP11−285F16.1
CWF19L1
LINC00263
HIF1AN
SNORA12
RNU6−422P
RP11−34D15.2
SLC22A20
NEAT1
SCYL1
MIR4690
AP5B1
CTSW
EIF1AD
snoU13
POLA2
FRMD8
MALAT1
PCNXL3
OVOL1
FIBP
SART1
PACS1
CDC42EP2
AP000769.1
KCNK7
KAT5
CFL1
TSGA10IP
SF3B2
DPF2
AP000769.7
SIPA1
RP11−770G2.4
DRAP1
CST6
TIGD3
LTBP3
MIR4489
RP11−770G2.5
BANF1
RP11−867O8.5
SSSCA1−AS1
RNASEH2C
SNX32
CATSPER1
SLC25A45
SSSCA1
RN7SL309P
MUS81
GAL3ST3
FAM89B
RP11−770G2.2
FOSL1
RP11−1167A19.2
7 genes omitted
CLEC2B
CLEC12B
OLR1
KLRD1
KLRK1
EIF2S3L
STYK1
TAS2R7
RP11−290C10.1
CLEC1A
GABARAPL1
RP11−277P12.20
KLRAP1
YBX3
PRR4
KLRF2
CLEC1B
CLEC7A
RP11−277P12.10
RP11−291B21.2
RP13−81N3.2
CLEC2A
CLEC9A
TMEM52B
RP11−277P12.9
MAGOHB
TAS2R8
AC091814.2
RN7SKP161
KLRC4−KLRK1
TAS2R9
AC091814.3
RP11−656E20.5
KLRC4
TAS2R10
CLEC12A
KLRC3
RP11−133L14.5
NKG2−E
2 genes omitted

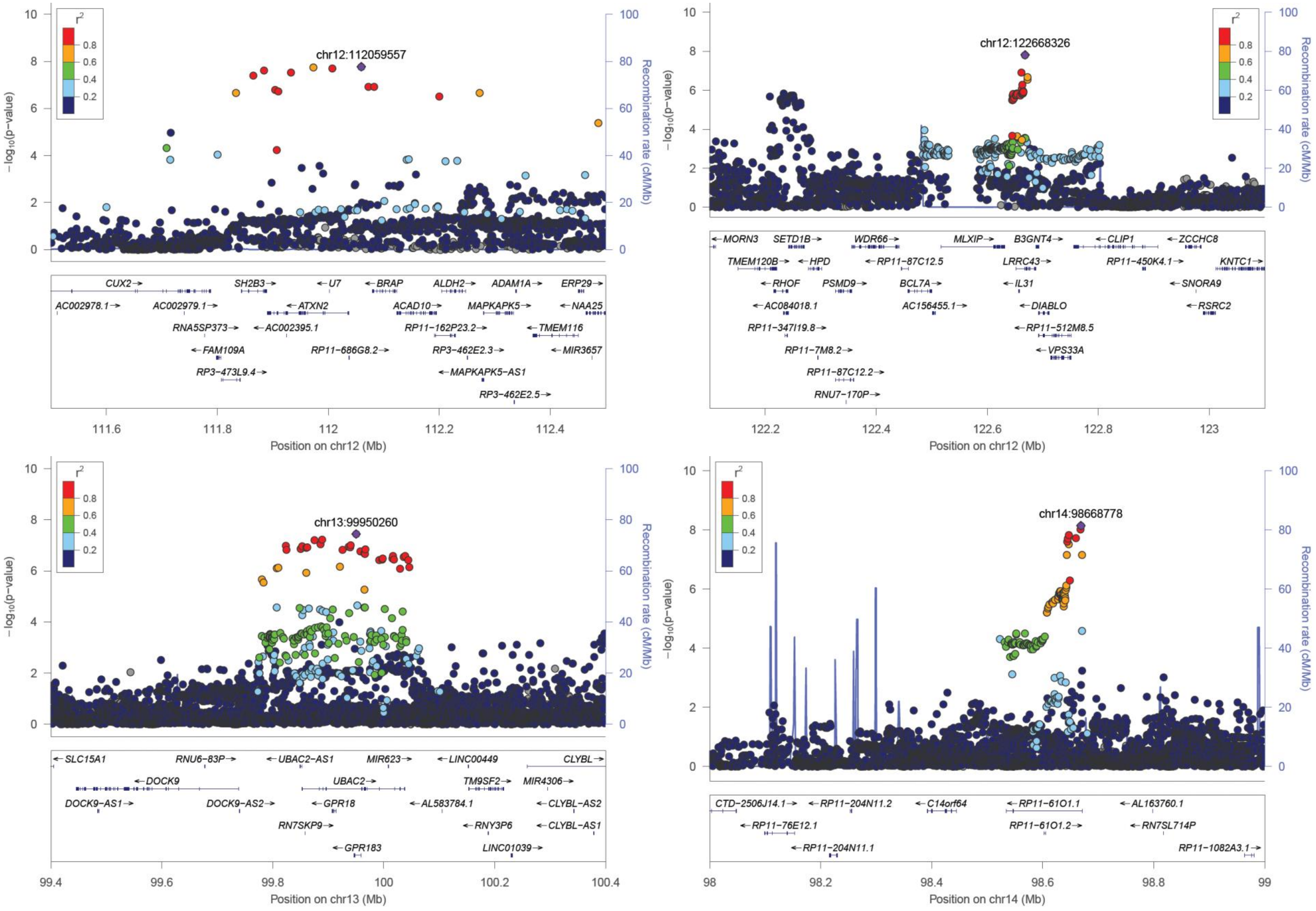

chr12:112059557
r2
0.8
0.6
0.4
0.2
−log10(p−value)
Recombination rate (cM/Mb)
CUX2→
SH2B3→
←U7
←BRAP
ALDH2→
ADAM1A→
ERP29→
AC002978.1→
AC002979.1→
←ATXN2
ACAD10→
MAPKAPK5→
←NAA25
RNA5SP373→
←AC002395.1
RP11−162P23.2→
←TMEM116
←FAM109A
RP11−686G8.2→
RP3−462E2.3→
←MIR3657
RP3−473L9.4→
←MAPKAPK5−AS1
RP3−462E2.5→
111.6
111.8
112
112.2
112.4
Position on chr12 (Mb)
chr12:122668326
←MORN3
SETD1B→
WDR66→
MLXIP→
B3GNT4→
←CLIP1
←ZCCHC8
TMEM120B→
←HPD
←RP11−87C12.5
LRRC43→
RP11−450K4.1→
KNTC1→
←RHOF
PSMD9→
BCL7A→
←IL31
←SNORA9
←AC084018.1
AC156455.1→
←DIABLO
←RSRC2
RP11−347I19.8→
←RP11−512M8.5
RP11−7M8.2→
←VPS33A
RP11−87C12.2→
RNU7−170P→
122.2
122.4
122.6
122.8
123
Position on chr12 (Mb)
chr13:99950260
←SLC15A1
RNU6−83P→
←UBAC2−AS1
MIR623→
←LINC00449
CLYBL→
←DOCK9
UBAC2→
TM9SF2→
MIR4306→
DOCK9−AS1→
DOCK9−AS2→
←GPR18
←AL583784.1
←CLYBL−AS2
RN7SKP9→
←RNY3P6
←CLYBL−AS1
←GPR183
LINC01039→
99.4
99.6
99.8
100
100.2
100.4
Position on chr13 (Mb)
chr14:98668778
CTD−2506J14.1→
←RP11−204N11.2
←C14orf64
←RP11−61O1.1
←AL163760.1
←RP11−76E12.1
RP11−61O1.2→
←RN7SL714P
←RP11−204N11.1
RP11−1082A3.1→
98
98.2
98.4
98.6
98.8
99
Position on chr14 (Mb)

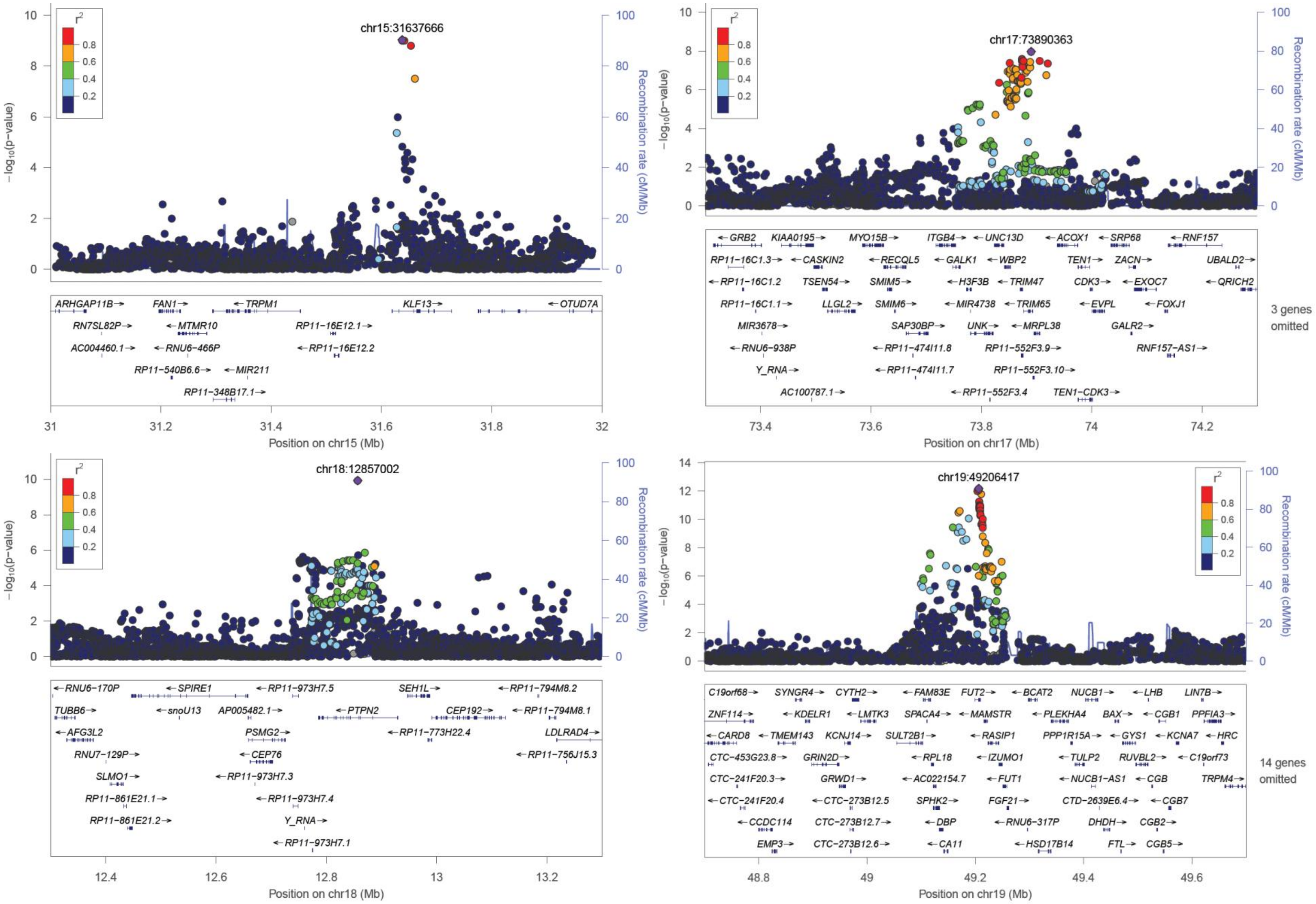
chr15:31637666
−log10(p−value)
Recombination rate (cM/Mb)
Position on chr15 (Mb)
ARHGAP11B FAN1 TRPM1 KLF13 OTUD7A
RN7SL82P MTMR10 RP11−16E12.1
AC004460.1 RNU6−466P RP11−16E12.2
RP11−540B6.6 MIR211
RP11−348B17.1
chr17:73890363
Position on chr17 (Mb)
GRB2 KIAA0195 MYO15B ITGB4 UNC13D ACOX1 SRP68 RNF157
RP11−16C1.3 CASKIN2 RECQL5 GALK1 WBP2 TEN1 ZACN UBALD2
RP11−16C1.2 TSEN54 SMIM5 H3F3B TRIM47 CDK3 EXOC7 QRICH2
RP11−16C1.1 LLGL2 SMIM6 MIR4738 TRIM65 EVPL FOXJ1
MIR3678 SAP30BP UNK MRPL38 GALR2
RNU6−938P RP11−474I11.8 RP11−552F3.9 RNF157−AS1
Y_RNA RP11−474I11.7 RP11−552F3.10
AC100787.1 RP11−552F3.4 TEN1−CDK3
3 genes omitted
chr18:12857002
Position on chr18 (Mb)
RNU6−170P SPIRE1 RP11−973H7.5 SEH1L RP11−794M8.2
TUBB6 snoU13 AP005482.1 PTPN2 CEP192 RP11−794M8.1
AFG3L2 PSMG2 RP11−773H22.4 LDLRAD4
RNU7−129P CEP76 RP11−756J15.3
SLMO1 RP11−973H7.3
RP11−861E21.1 RP11−973H7.4
RP11−861E21.2 Y_RNA
RP11−973H7.1
chr19:49206417
Position on chr19 (Mb)
C19orf68 SYNGR4 CYTH2 FAM83E FUT2 BCAT2 NUCB1 LHB LIN7B
ZNF114 KDELR1 LMTK3 SPACA4 MAMSTR PLEKHA4 BAX CGB1 PPFIA3
CARD8 TMEM143 KCNJ14 SULT2B1 RASIP1 PPP1R15A GYS1 KCNA7 HRC
CTC−453G23.8 GRIN2D RPL18 IZUMO1 TULP2 RUVBL2 C19orf73
CTC−241F20.3 GRWD1 AC022154.7 FUT1 NUCB1−AS1 CGB TRPM4
CTC−241F20.4 CTC−273B12.5 SPHK2 FGF21 CTD−2639E6.4 CGB7
CCDC114 CTC−273B12.7 DBP RNU6−317P DHDH CGB2
EMP3 CTC−273B12.6 CA11 HSD17B14 FTL CGB5
14 genes omitted

Supplementary Figure 5. Regional association plots for 16 new psoriasis loci identified in this cohort. The y-axis represents the –log10 p-values of the meta-analysis results (left) and recombination rate (right). Build 37 (GRCh37) was used to generate these plots

.

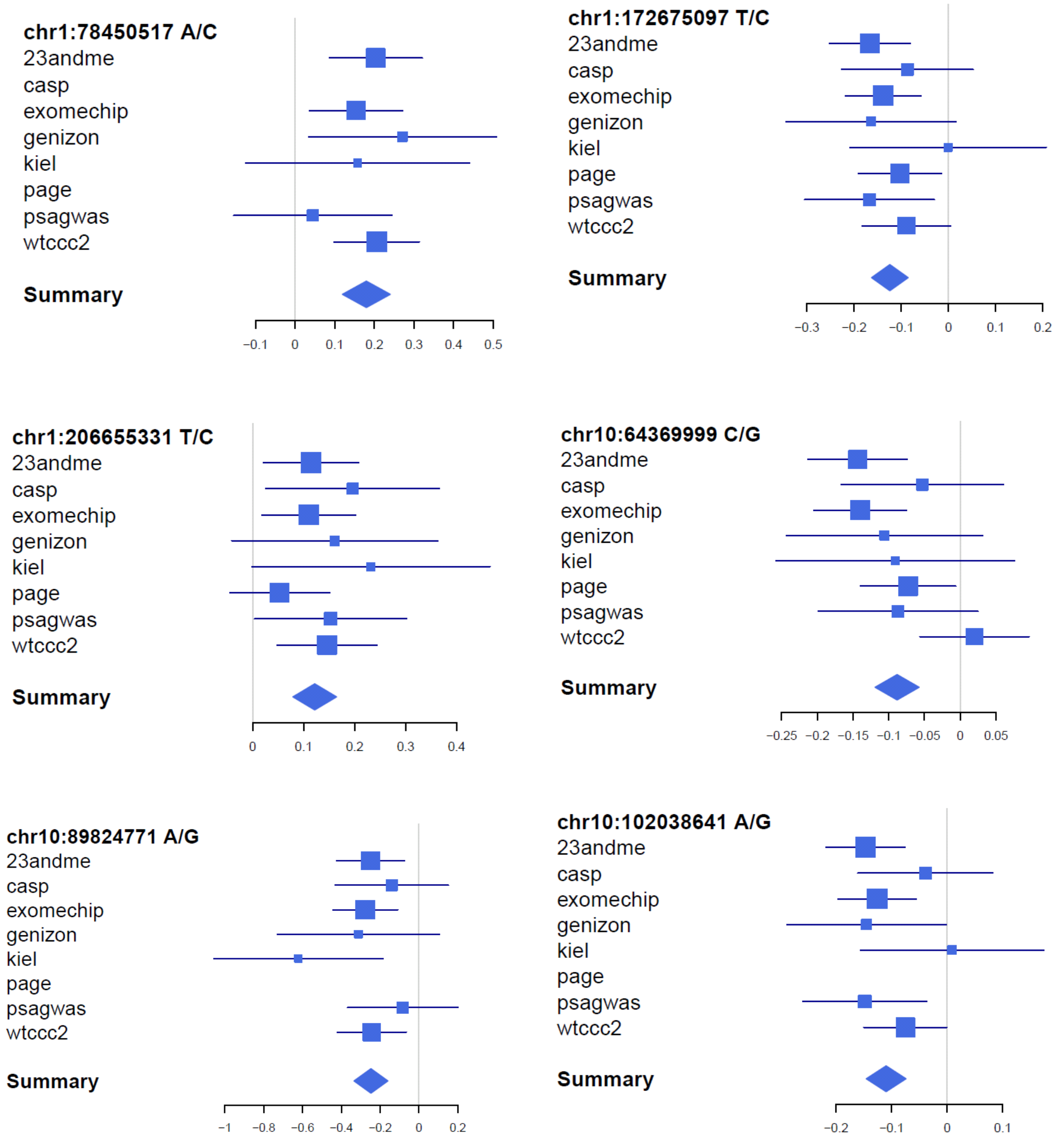

chr1:78450517 A/C
23andme
casp
exomechip
genizon
kiel
page
psagwas
wtccc2
Summary
−0.1 0 0.1 0.2 0.3 0.4 0.5
chr1:172675097 T/C
23andme
casp
exomechip
genizon
kiel
page
psagwas
wtccc2
Summary
−0.3 −0.2 −0.1 0 0.1 0.2
chr1:206655331 T/C
23andme
casp
exomechip
genizon
kiel
page
psagwas
wtccc2
Summary
0 0.1 0.2 0.3 0.4
chr10:64369999 C/G
23andme
casp
exomechip
genizon
kiel
page
psagwas
wtccc2
Summary
−0.25 −0.2 −0.15 −0.1 −0.05 0 0.05
chr10:89824771 A/G
23andme
casp
exomechip
genizon
kiel
page
psagwas
wtccc2
Summary
−1 −0.8 −0.6 −0.4 −0.2 0 0.2
chr10:102038641 A/G
23andme
casp
exomechip
genizon
kiel
page
psagwas
wtccc2
Summary
−0.2 −0.1 0 0.1

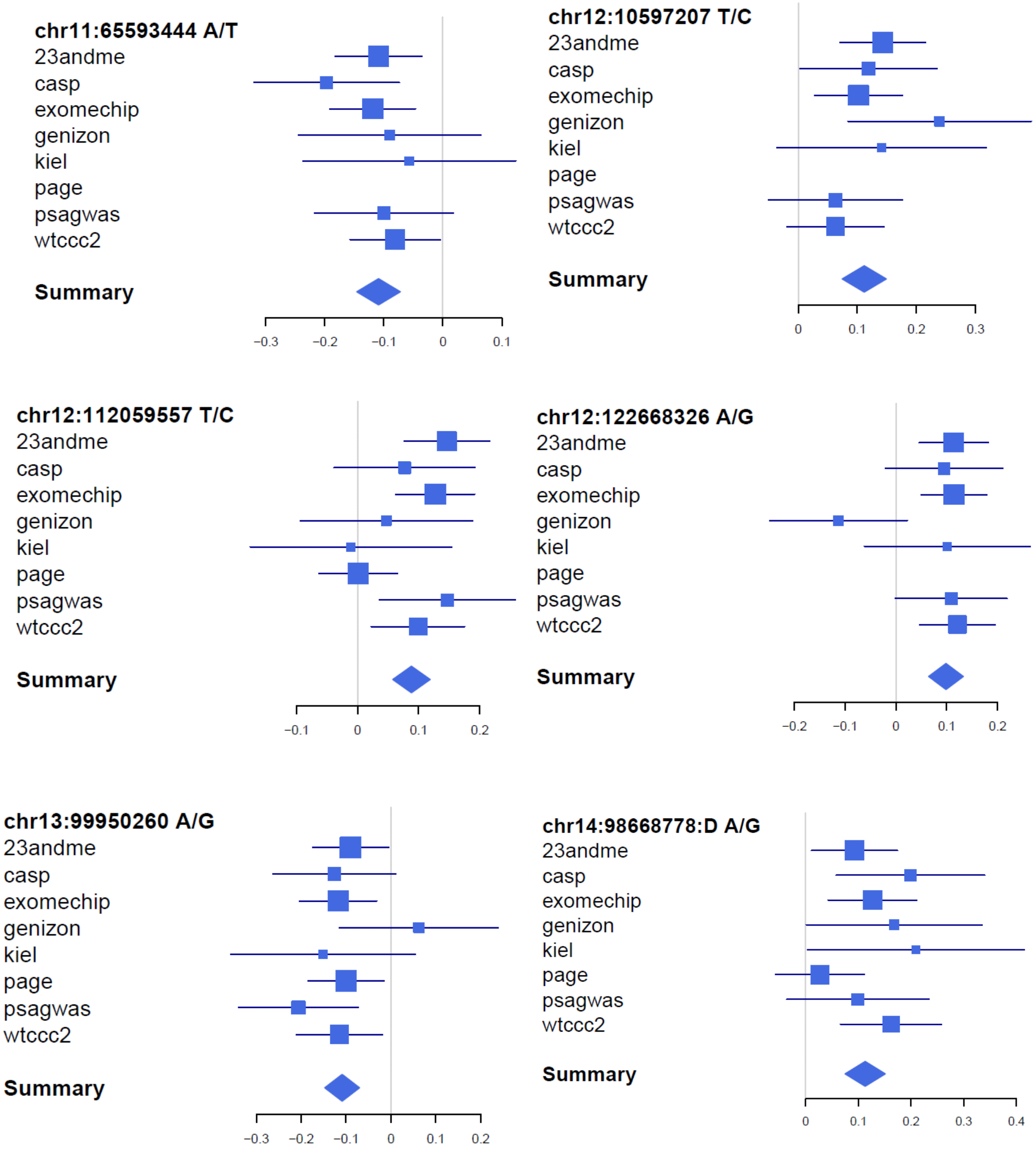
chr11:65593444 A/T
23andme
casp
exomechip
genizon
kiel
page
psagwas
wtccc2
Summary
−0.3
−0.2
−0.1
0
0.1
chr12:10597207 T/C
23andme
casp
exomechip
genizon
kiel
page
psagwas
wtccc2
Summary
0
0.1
0.2
0.3
chr12:112059557 T/C
23andme
casp
exomechip
genizon
kiel
page
psagwas
wtccc2
Summary
−0.1
0
0.1
0.2
chr12:122668326 A/G
23andme
casp
exomechip
genizon
kiel
page
psagwas
wtccc2
Summary
−0.2
−0.1
0
0.1
0.2
chr13:99950260 A/G
23andme
casp
exomechip
genizon
kiel
page
psagwas
wtccc2
Summary
−0.3
−0.2
−0.1
0
0.1
0.2
chr14:98668778:D A/G
23andme
casp
exomechip
genizon
kiel
page
psagwas
wtccc2
Summary
0
0.1
0.2
0.3
0.4

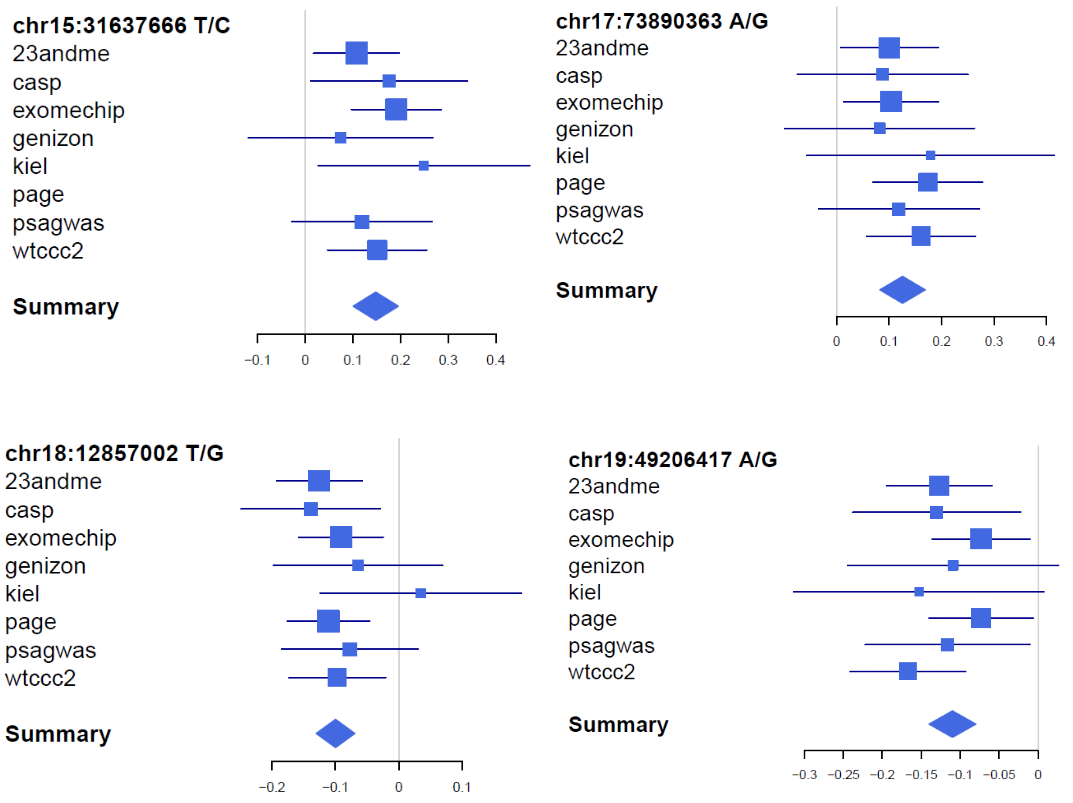

Supplementary Figure 6. Forest plots for the 16 novel loci to illustrate the betas and 95% confidence intervals for each cohort and the meta-analysis. The effect and non-effect alleles are shown after the marker positions for each locus. The x-axes denote the effect sizes.

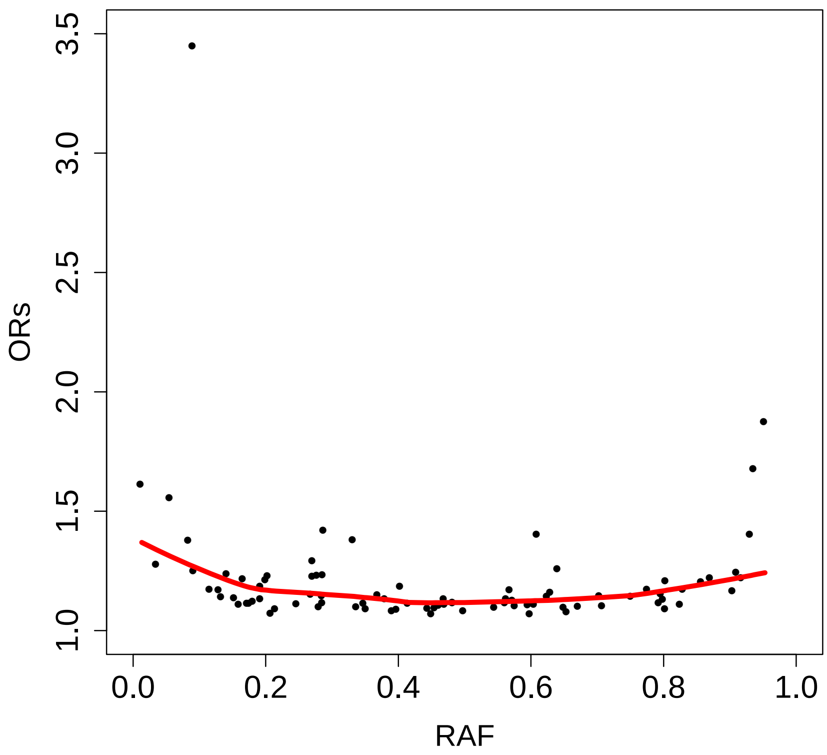

Supplementary Figure 7. Effect sizes (odds ratios, OR) versus risk allele frequencies (RAF) for all loci.

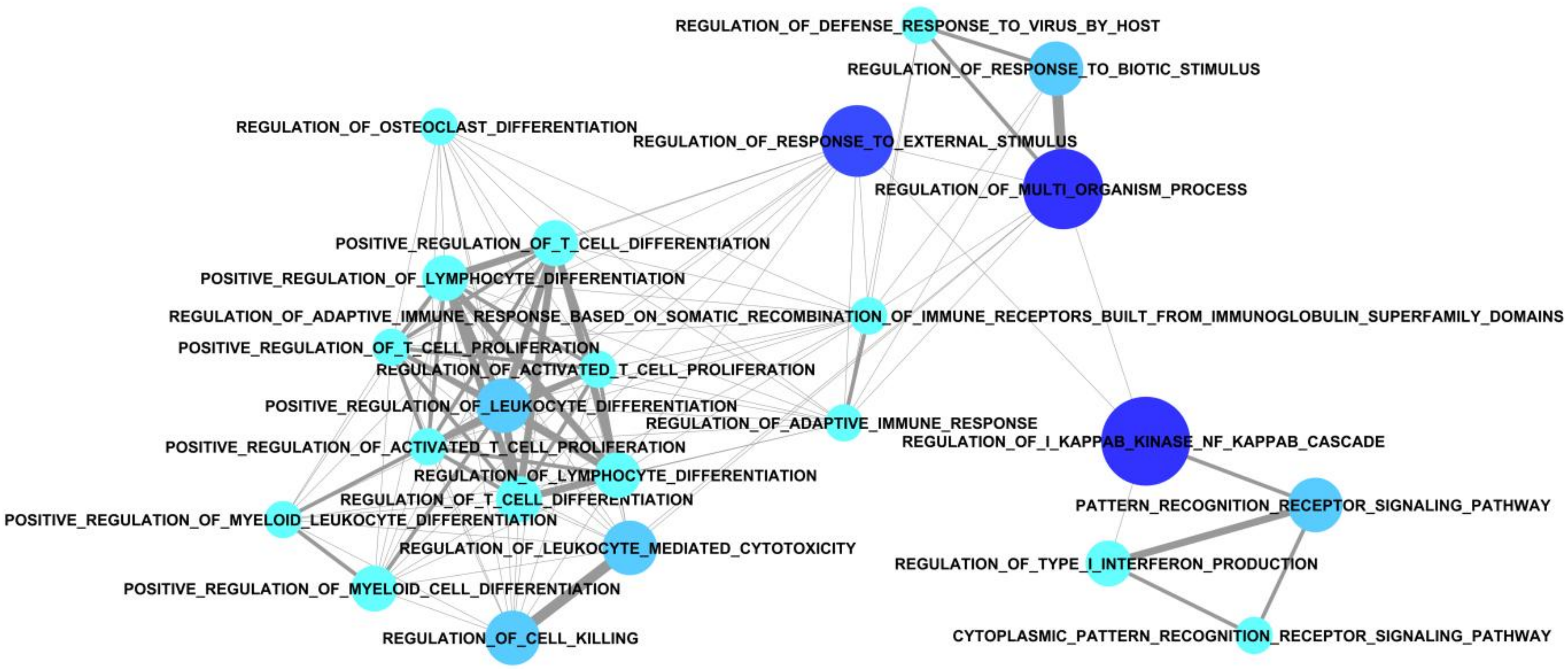

Supplementary Figure 8. Enriched functions with genes overlapped with other enriched function. This is the reproduction of Figure 2a with node labels. Enriched functions (nodes) among the associated loci identified using MEAGA. For illustration purposes, only functions with at least four genes overlapping with other enriched functions are shown. The size of the nodes and the width of the edges correlate with the number of overlapped disease-loci and the number of shared disease-loci, respectively. Blue color represents higher number of overlapped loci and red color represents lower number of overlapped loci.

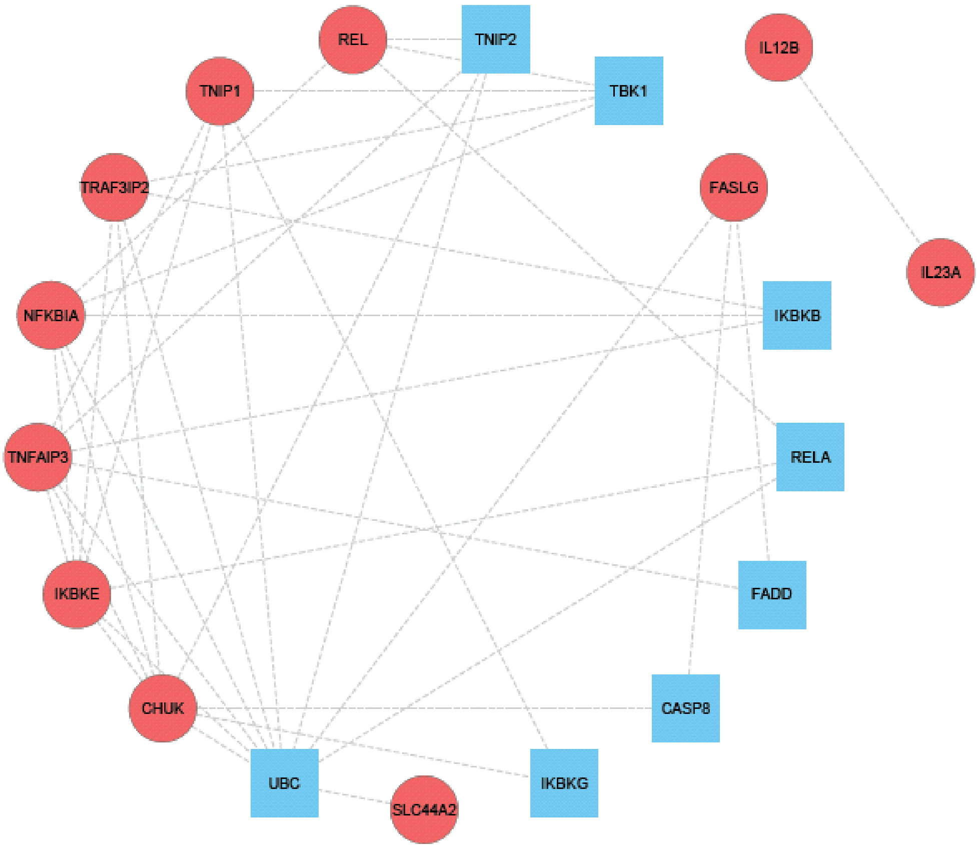

Supplementary Figure 9. Genes from the enriched function ("Regulation of I-κB kinase/NFκB cascade") which has one of the highest number of associated loci-overlapped genes. Red color represents genes from the associated loci, and blue color represents genes from the enriched function that have interactions with associated loci.

Supplementary Table

Supplementary Table 1. Datasets used in the meta-analysis. The number of cohort-defined cases/controls after quality control are listed.

Cohort	Number of defined cases after QC	Number of defined controls after QC	Effective Sample size*	Number of well-imputed markers ($r^2 \geq 0.7$)	Dataset Genomic Inflation factor
**PsA GWAS**	1,430	1,417	2,847	10,752,852	1.03
**CASP GWAS[1]**	1,352	1,389	2,741	8,748,985	1.02
**Kiel GWAS[2]**	464	1,135	1,317	8,920,790	1.05
**Genizon GWAS[2]**	761	993	1,723	9,046,475	1.02
**WTCCC2 GWAS[3]**	2,178	5,175	6,131	10,582,016	1.04
**Exomechip w/ GWAS content**	3,863	4,028	7,888	8,394,884	1.02
**PAGE Immunochip[4]**	3,181	7,406	8,901	757,723	1.02
**23andMe GWAS**	*5,803*[#]	*265,226*[#]	7,950	11,134,041	1.05
**TOTAL**	19,032	286,769	39,498		

[#]Estimated numbers of true cases and controls based on self-reported data and misclassification estimation (see Methods)

*The effective sample size for each cohort with dermatologist-defined cases was computed as: $4/(1/N_{case}+1/N_{control})$; the 23andMe GWAS uses self-reported information to define cases, and we determined the effective sample size using misclassification adjustment simulated data and the asymptotic relative efficiency approach (see **Methods**).

Supplementary Table 2. Summary statistics for previously identified loci. EA: effect allele; NEA: non-effect allele. Direction represents whether the EA increases (+) or decreases (-) the risk of psoriasis.

Chr	Position	EA	NEA	Beta	p-value	Direction
1	8286009	a	g	0.1208	4.26E-10	++++++++
1	24518206	t	c	-0.1512	4.82E-16	-?------
1	25291010	a	t	-0.1407	2.93E-19	--------
1	67713346	t	c	0.3904	2.46E-29	++++++++
1	69788482	t	g	-0.0682	0.0001644	-----+--
1	152591953	c	g	0.1771	1.63E-27	++++++++
1	197757846	c	g	-0.1005	3.53E-07	--------
2	61072183	a	t	0.1665	2.33E-26	++++++++
2	62560332	a	g	0.1157	2.78E-13	++++++++
2	163167746	c	g	0.3105	1.07E-24	++++++++
3	16996623	a	g	-0.1198	2.22E-14	--------
3	101647309	t	g	-0.1061	8.37E-11	--------
3	189662658	a	g	0.1394	4.95E-09	++++++?+
5	40370724	a	c	-0.1215	1.41E-05	--------
5	96118852	c	g	-0.1444	4.37E-18	--------
5	131996445	a	g	-0.196	4.91E-23	--------
5	150469973	a	g	0.4863	9.79E-59	++++++++
5	158829527	a	t	-0.5294	2.92E-84	--------
6	577820	a	g	0.091	4.60E-09	-+++++++
6	20689945	a	g	0.1676	6.40E-19	++++++++
6	30916259	a	g	1.1598	5.58e-325	++++++++
6	111929862	t	g	0.3475	1.93E-42	++++++++
6	138197824	t	c	-0.1765	1.04E-25	--------
6	159506600	t	c	-0.1109	5.82E-12	---+----
7	37385365	a	g	0.0995	1.91E-10	++++++++
9	32523737	t	c	0.0918	2.84E-09	++++++++
9	110792282	t	c	0.1004	9.15E-10	++++++++
10	75601596	t	c	-0.127	1.90E-15	--------
10	81043743	a	g	0.1062	1.72E-11	++++++++
11	64053157	t	g	0.0953	1.10E-08	++++++++
11	109962432	a	g	0.1239	9.32E-16	++++++++
11	128391937	t	c	0.1426	8.69E-15	++++++++
12	56741228	a	g	-0.3421	3.79E-25	--------
13	40745693	a	g	-0.2019	1.21E-07	------?-
13	45321731	a	c	-0.0972	1.18E-08	--------
14	35839236	c	g	0.1521	1.92E-19	++++++++
16	11354091	a	g	0.1367	9.84E-16	++++++++

16	31021078	t	c	0.1275	1.13E-15	+++-++++
17	26124908	a	g	0.2118	9.23E-23	++++++++
17	40536396	t	c	-0.1024	1.84E-10	--------
17	78175483	t	c	0.1015	1.27E-08	++++++?+
18	51816394	t	c	0.1214	1.04E-12	++++++++
19	10463118	c	g	-0.6762	3.39E-43	-?-?----
19	10886206	t	g	-0.0994	2.00E-10	--------
20	48574454	a	g	-0.1462	6.52E-21	--------
21	36488822	t	c	-0.1181	6.10E-08	---+----
22	21974703	c	g	0.1377	6.81E-13	++++++++

Supplementary Table 3. Detailed information regarding the new loci identified in this study. The “Meta p without 23andMe” column shows the p-values of the meta-analysis including all but the 23andMe dataset. The “Meta P value (wo/adj)” and “Meta P value (w/adj)” illustrate the meta-analysis results without and with the Duffy’s adjustment, respectively. The “Direction” column indicates the direction of the meta-analysis-identified risk allele in each dataset (P: PsA GWAS; C: CASP GWAS; K: Kiel GWAS; G: Genizon GWAS; W: WTCCC2; E: Exomechip; I: PAGE Immunochip; M: 23andMe GWAS).

Chr	Pos	Marker	Meta p without 23andMe	Meta P value (wo/adj)	Meta OR (wo/adj)	Meta P value (w/adj)	Heterogeneity p-value (w/adj)	OR(w/adj)	Direction (PCKGWEIM)	Nearby genes
1	78450517	rs34517439	1.19E-06	4.95E-08	1.10	4.43E-09	0.72	1.18	+?++++?+	*FUBP1*
1	172675097	rs12118303	2.07E-07	7.17E-10	1.08	3.02E-10	0.8	1.12	++++++++	*FASLG*
1	206655331	rs41298997	4.01E-07	1.09E-06	1.07	2.37E-08	0.78	1.13	++++++++	*IKBKE*
10	64369999	rs2944542	1.35E-05	3.27E-09	1.06	1.76E-08	7.8E-02	1.08	++++-+++	*ZNF365*
10	89824771	rs76959677	1.37E-06	7.49E-07	1.14	2.75E-08	0.59	1.28	++++++?+	*PTEN, KLLN, SNORD74*
10	102038641	rs61871342	2.63E-06	1.93E-09	1.07	1.56E-09	0.44	1.10	++-+++?+	*CHUK*
11	65593444	rs118086960	4.65E-07	2.63E-07	1.06	6.89E-09	0.82	1.12	++++++?+	*CFL1, FIBP, FOSL1*
12	10597207	rs11053802	4.01E-06	9.09E-09	1.07	4.17E-09	0.49	1.11	++++++?+	*KLRK1, KLRC4*
12	112059557	rs11065979	1.38E-05	2.41E-09	1.06	1.67E-08	6.09E-02	1.08	++-+++++	*BRAP, MAPKAPK5*
12	122668326	rs11059675	2.77E-06	1.23E-07	1.06	1.50E-08	0.13	1.10	+++-++?+	*IL31*
13	99950260	rs9513593	2.75E-07	3.12E-06	1.06	3.60E-08	0.29	1.12	+++-++++	*UBAC2, RN7SKP9*
14	98668778	rs142903734	7.08E-08	1.08E-06	1.06	7.15E-09	0.34	1.12	++++++++	*RP11-61O1.1*
15	31637666	rs28624578	4.15E-08	8.00E-07	1.07	9.22E-10	0.80	1.18	++++++?+	*KLF13*
17	73890363	rs55823223	8.39E-08	1.61E-06	1.07	1.06E-08	0.94	1.15	++++++++	*TRIM47, TRIM65*
18	12857002	rs559406	5.72E-08	7.05E-10	1.06	1.19E-10	0.75	1.10	++-+++++	*PTPN2*
19	49206417	rs492602	4.24E-10	2.23E-11	1.07	6.57E-13	0.62	1.11	++++++++	*FUT2*

Supplementary Table 4. Information for the independent signals used to construct Figure 1 and used for the regulatory element enrichment analysis. RA: risk allele; NRA: non-risk allele.

	RA	NRA	OR	MAF
**chr1:8273177**	G	T	1.15	0.29
**chr1:24518643**	G	A	1.21	0.14
**chr1:25297184**	A	G	1.14	0.47
**chr1:67702526**	T	G	1.68	0.06
**chr1:67705574**	T	C	1.12	0.40
**chr1:67619259**	G	T	1.11	0.34
**chr1:69788482**	G	T	1.12	0.25
**chr1:152593549**	T	C	1.26	0.36
**chr2:61083506**	T	C	1.17	0.37
**chr2:62552321**	A	G	1.14	0.38
**chr2:163110536**	G	A	1.41	0.39
**chr2:163128824**	T	C	1.30	0.27
**chr3:16996035**	G	A	1.12	0.48
**chr3:101663555**	A	G	1.14	0.20
**chr5:40370724**	C	A	1.17	0.09
**chr5:96120170**	G	A	1.24	0.29
**chr5:96290647**	T	C	1.09	0.39
**chr5:131996669**	C	T	1.16	0.20
**chr5:150466690:D**	G	A	1.56	0.06
**chr5:158826310**	G	C	1.22	0.20
**chr5:158748012**	C	T	1.21	0.19
**chr5:158829527**	T	A	1.26	0.09
**chr5:158812417**	C	T	1.15	0.29
**chr5:158871681**	T	A	1.23	0.08
**chr5:158839745**	T	C	1.10	0.35
**chr6:515393**	C	T	1.12	0.42
**chr6:31251924**	T	C	3.45	0.09
**chr6:31353651:I**	C	A	1.39	0.33
**chr6:29803526**	T	G	1.43	0.29
**chr6:31249217**	A	G	1.23	0.20
**chr6:31431820**	C	T	1.12	0.17
**chr6:31457362**	C	A	1.19	0.20
**chr6:29555585**	A	G	1.15	0.25
**chr6:32392757**	A	G	1.62	0.01
**chr6:31264823**	A	G	1.22	0.17
**chr6:29923554**	T	C	1.19	0.40
**chr6:31553151:D**	A	G	1.23	0.13

**chr6:111929862**	T	G	1.38	0.09
**fachr6:111580561**	C	A	1.10	0.28
**chr6:138197824**	C	T	1.23	0.27
**chr6:159492044**	T	C	1.18	0.13
**chr7:37386237**	A	C	1.13	0.44
**chr9:32523737**	T	C	1.11	0.40
**chr9:110792282**	T	C	1.15	0.37
**chr10:75599127**	A	G	1.12	0.44
**chr10:81032532**	A	G	1.11	0.42
**chr11:64115137**	C	T	1.12	0.39
**chr11:109973130**	C	A	1.18	0.43
**chr11:128406438**	A	G	1.12	0.48
**chr12:56744422**	G	A	1.41	0.07
**chr12:56497903**	T	C	1.10	0.35
**chr14:35839236**	C	G	1.24	0.28
**chr14:35840257**	C	T	1.16	0.27
**chr16:11365500**	C	T	1.11	0.29
**chr16:31006972**	T	C	1.16	0.37
**chr17:26124908**	A	G	1.24	0.14
**chr17:26096473**	A	C	1.12	0.35
**chr17:40563017**	A	G	1.14	0.15
**chr17:78178830**	A	G	1.09	0.50
**chr18:51791375**	C	G	1.12	0.29
**chr19:10463118**	G	C	1.88	0.05
**chr19:10469975**	A	C	1.25	0.09
**chr19:10886206**	G	T	1.14	0.44
**chr20:48582769**	A	C	1.13	0.43
**chr20:48642702**	T	C	1.12	0.16
**chr22:21925017**	G	A	1.14	0.19
**chr1:197671115**	T	C	1.10	0.22
**chr3:189615475**	C	A	1.10	0.20
**chr6:20678430**	C	G	1.18	0.22
**chr13:40333369**	T	C	1.08	0.34
**chr13:45334194**	C	T	1.09	0.40
**chr21:36470865**	T	C	1.08	0.21
**chr1:78450517**	A	C	1.18	0.12
**chr1:172675097**	C	T	1.12	0.17
**chr1:206655331**	T	C	1.13	0.18
**chr10:64369999**	G	C	1.08	0.40
**chr10:89824771**	G	A	1.28	0.04
**chr10:102038641**	G	A	1.10	0.45

**chr11:65593444**	T	A	1.12	0.47
**chr12:10597207**	T	C	1.11	0.33
**chr12:112059557**	T	C	1.08	0.45
**chr12:122668326**	A	G	1.10	0.46
**chr13:99950260**	G	A	1.12	0.18
**chr14:98668778:D**	A	G	1.12	0.21
**chr15:31637666**	T	C	1.18	0.17
**chr17:73890363**	A	G	1.15	0.13
**chr18:12857002**	G	T	1.10	0.45
**chr19:49206417**	G	A	1.11	0.46

Supplementary Table 5. Heritability explained (%) by known loci using GCTA. Heritability explained by psoriasis loci determined by (the phenotypic variance explained by psoriasis loci) / (phenotypic variance explained by genotyped markers in the corresponding cohort).

Psoriasis Prevalence	Cohort		
	**PAGE**	**Exomechip (full)**	**WTCCC2**
**2%**	13/24.7 = 52.6	11/46.4 = 23.7	16.3/74.2 = 22
**2.5%**	13.8/26.2 = 52.7	11.7/49.2 = 23.8	17.3/78.7 = 22
**3%**	14.5/27.6 = 52.5	12.3/51.8 = 23.7	18.2/82.8 = 22

Supplementary Table 6. Enriched functions identified by MEAGA.

Enriched Functions	# genes in functions	# of overlapped genes	# of connected graph	pval	False Discovery Rate	Genes
POSITIVE_REGULATION_OF_CELL_FATE_COMMITMENT	6	3	1	≤2.00E-05	≤2.08E-02	IL12B;IL23A;IL23R
REGULATION_OF_T_HELPER_1_TYPE_IMMUNE_RESPONSE	20	3	1	≤2.00E-05	≤2.08E-02	IL12B;IL23A;IL23R
NEGATIVE_REGULATORS_OF_RIG_I_MDA5_SIGNALING	22	4	1	≤2.00E-05	≤2.08E-02	DDX58;IFIH1;TNFAIP3;IKBKE
REGULATION_OF_TYPE_I_INTERFERON_PRODUCTION	70	6	1	≤2.00E-05	≤2.08E-02	CHUK;DDX58;IFIH1;IKBKE;NFKBIA;TNFAIP3
POSITIVE_REGULATION_OF_NATURAL_KILLER_CELL_ACTIVATION	18	4	2	≤2.00E-05	≤2.08E-02	IL12B;IL23A;IL23R;STAT5A
REGULATION_OF_NK_T_CELL_ACTIVATION	6	3	1	≤2.00E-05	≤2.08E-02	IL12B;IL23A;IL23R
POSITIVE_REGULATION_OF_MYELOID_CELL_DIFFERENTIATION	63	6	3	≤2.00E-05	≤2.08E-02	ETS1;IL12B;IL23A;IL23R;RUNX1;STAT5A
POSITIVE_REGULATION_OF_T_HELPER_1_TYPE_IMMUNE_RESPONSE	9	3	1	≤2.00E-05	≤2.08E-02	IL12B;IL23A;IL23R
REGULATION_OF_ACTIVATION_OF_JANUS_KINASE_ACTIVITY	7	3	1	≤2.00E-05	≤2.08E-02	IL12B;IL23A;IL23R
POSITIVE_REGULATION_OF_OSTEOCLAST_DIFFERENTIATION	15	3	1	≤2.00E-05	≤2.08E-02	IL12B;IL23A;IL23R
POSITIVE_REGULATION_OF_ACTIVATION_OF_JANUS_KINASE_ACTIVITY	7	3	1	≤2.00E-05	≤2.08E-02	IL12B;IL23A;IL23R
REGULATION_OF_LEUKOCYTE_MEDIATED_CYTOTOXICITY	33	7	4	≤2.00E-05	≤2.08E-02	ICAM1;IL12B;IL23A;IL23R;KLRK1;NOS2;STAT5A
REGULATION_OF_ACTIVATION_OF_JAK2_KINASE_ACTIVITY	6	3	1	≤2.00E-05	≤2.08E-02	IL12B;IL23A;IL23R
REGULATION_OF_CELL_FATE_COMMITMENT	25	3	1	≤2.00E-05	≤2.08E-02	IL12B;IL23A;IL23R
REGULATION_OF_TYROSINE_PHOSPHORYLATION_OF_STAT5_PROTEIN	18	4	2	≤2.00E-05	≤2.08E-02	IL12B;IL23A;IL23R;IL4
POSITIVE_REGULATION_OF_INTERLEUKIN_17_PRODUCTION	11	3	1	≤2.00E-05	≤2.08E-02	IL12B;IL23A;IL23R
REGULATION_OF_T_HELPER_17_TYPE_IMMUNE_RESPONSE	7	4	2	≤2.00E-05	≤2.08E-02	IL12B;IL23A;IL23R;IL4
POSITIVE_REGULATION_OF_T_HELPER_17_TYPE_IMMUNE_RESPONSE	6	3	1	≤2.00E-05	≤2.08E-02	IL12B;IL23A;IL23R
REGULATION_OF_MEMORY_T_CELL_DIFFERENTIATION	7	3	1	≤2.00E-05	≤2.08E-02	IL12B;IL23A;IL23R
REGULATION_OF_INTERLEUKIN_10_PRODUCTION	29	4	2	≤2.00E-05	≤2.08E-02	IL12B;IL23A;IL23R;IL4
POSITIVE_REGULATION_OF_GRANULOCYTE_MACROPHAGE_COLONY_STIMULATING_FACTOR_PRODUCTION	6	3	1	≤2.00E-05	≤2.08E-02	IL12B;IL23A;IL23R
POSITIVE_REGULATION_OF_MEMORY_T_CELL_DIFFERENTIATION	5	3	1	≤2.00E-05	≤2.08E-02	IL12B;IL23A;IL23R

REGULATION_OF_INTERLEUKIN_17_PRODUCTION	18	3	1	≤2.00E-05	≤2.08E-02	IL12B;IL23A;IL23R
REGULATION_OF_DEFENSE_RESPONSE_TO_VIRUS_BY_HOST	23	5	3	≤2.00E-05	≤2.08E-02	DDX58;IL12B;IL23A;IL23R;TNFAIP3
POSITIVE_REGULATION_OF_ACTIVATED_T_CELL_PROLIFERATION	18	5	3	≤2.00E-05	≤2.08E-02	IL12B;IL23A;IL23R;IL4;STAT5A
POSITIVE_REGULATION_OF_ACTIVATION_OF_JAK2_KINASE_ACTIVITY	6	3	1	≤2.00E-05	≤2.08E-02	IL12B;IL23A;IL23R
NEGATIVE_REGULATION_OF_INTERLEUKIN_10_PRODUCTION	7	3	1	≤2.00E-05	≤2.08E-02	IL12B;IL23A;IL23R
POSITIVE_REGULATION_OF_NK_T_CELL_ACTIVATION	5	3	1	≤2.00E-05	≤2.08E-02	IL12B;IL23A;IL23R
CYTOPLASMIC_PATTERN_RECOGNITION_RECEPTOR_SIGNALING_PATHWAY	31	5	1	≤2.00E-05	≤2.08E-02	CHUK;DDX58;IFIH1;NFKBIA;TNFAIP3
REGULATION_OF_CELL_KILLING	36	7	4	≤2.00E-05	≤2.08E-02	ICAM1;IL12B;IL23A;IL23R;KLRK1;NOS2;STAT5A
POSITIVE_REGULATION_OF_ALPHA_BETA_T_CELL_ACTIVATION	44	3	1	≤2.00E-05	≤2.08E-02	IL12B;IL23A;IL23R
CYTOSOLIC_DNA_SENSING_PATHWAY	56	4	1	≤2.00E-05	≤2.08E-02	CHUK;DDX58;NFKBIA;IKBKE
REGULATION_OF_MULTI_ORGANISM_PROCESS	216	10	2	≤2.00E-05	≤2.08E-02	DDX58;ELMO1;FYN;IL12B;IL23A;IL23R;ILF3;NOS2;TNFAIP3;TNIP1
REGULATION_OF_NATURAL_KILLER_CELL_ACTIVATION	24	4	2	≤2.00E-05	≤2.08E-02	IL12B;IL23A;IL23R;STAT5A
TRAF3_DEPENDENT_IRF_ACTIVATION_PATHWAY	12	3	1	≤2.00E-05	≤2.08E-02	DDX58;IFIH1;IKBKE
PATTERN_RECOGNITION_RECEPTOR_SIGNALING_PATHWAY	132	7	1	≤2.00E-05	≤2.08E-02	CHUK;DDX58;IFIH1;IKBKE;NFKBIA;TNFAIP3;TNIP1
DEFENSE_RESPONSE_TO_GRAM_NEGATIVE_BACTERIUM	18	4	2	≤2.00E-05	≤2.08E-02	IL12B;IL23A;IL23R;NOS2
POSITIVE_REGULATION_OF_NATURAL_KILLER_CELL_PROLIFERATION	6	3	1	≤2.00E-05	≤2.08E-02	IL12B;IL23A;IL23R
REGULATION_OF_ACTIVATED_T_CELL_PROLIFERATION	25	5	3	≤2.00E-05	≤2.08E-02	IL12B;IL23A;IL23R;IL4;STAT5A
POSITIVE_REGULATION_OF_TYROSINE_PHOSPHORYLATION_OF_STAT5_PROTEIN	16	4	2	≤2.00E-05	≤2.08E-02	IL12B;IL23A;IL23R;IL4
REGULATION_OF_GRANULOCYTE_MACROPHAGE_COLONY_STIMULATING_FACTOR_PRODUCTION	10	3	1	≤2.00E-05	≤2.08E-02	IL12B;IL23A;IL23R
REGULATION_OF_T_HELPER_17_CELL_DIFFERENTIATION	5	4	2	≤2.00E-05	≤2.08E-02	IL12B;IL23A;IL23R;IL4
POSITIVE_REGULATION_OF_TYROSINE_PHOSPHORYLATION_OF_STAT3_PROTEIN	27	3	1	≤2.00E-05	≤2.08E-02	IL12B;IL23A;IL23R
REGULATION_OF_TYROSINE_PHOSPHORYLATION_OF_STAT1_PROTEIN	12	3	1	≤2.00E-05	≤2.08E-02	IL12B;IL23A;IL23R
REGULATION_OF_NATURAL_KILLER_CELL_PROLIFERATION	7	3	1	≤2.00E-05	≤2.08E-02	IL12B;IL23A;IL23R
POSITIVE_REGULATION_OF_INTERLEUKIN_12_PRODUCTION	24	3	1	≤2.00E-05	≤2.08E-02	IL12B;IL23A;IL23R
POSITIVE_REGULATION_OF_DEFENSE_RESPONSE_TO_VIRUS_BY_HOST	15	4	2	≤2.00E-05	≤2.08E-02	DDX58;IL12B;IL23A;IL23R

REGULATION_OF_RESPONSE_TO_BIOTIC_STIMULUS	87	7	2	4.00E-05	3.19E-02	DDX58;ELMO1;FYN;IL12B;IL23A;IL23R;TNFAIP3
TRAF6_MEDIATED_IRF7_ACTIVATION	25	3	1	4.00E-05	3.19E-02	DDX58;IFIH1;IKBKE
REGULATION_OF_TYROSINE_PHOSPHORYLATION_OF_STAT3_PR OTEIN	32	4	2	4.00E-05	3.19E-02	IL12B;IL23A;IL23R;SOCS1
REGULATION_OF_ALPHA_BETA_T_CELL_DIFFERENTIATION	41	4	2	4.00E-05	3.19E-02	IL12B;IL23A;IL23R;IL4
POSITIVE_REGULATION_OF_LYMPHOCYTE_DIFFERENTIATION	65	6	3	4.00E-05	3.19E-02	BAD;IL12B;IL23A;IL23R;IL4;STAT5A
REGULATION_OF_T_HELPER_CELL_DIFFERENTIATION	22	4	2	4.00E-05	3.19E-02	IL12B;IL23A;IL23R;IL4
REGULATION_OF_RESPONSE_TO_EXTERNAL_STIMULUS	387	9	2	4.00E-05	3.19E-02	IL12B;IL23A;IL4;PLAU;PSMA6;PTEN;STAT5A;TNFAI P3;TNIP1
REGULATION_OF_ALPHA_BETA_T_CELL_ACTIVATION	60	4	2	4.00E-05	3.19E-02	IL12B;IL23A;IL23R;IL4
POSITIVE_REGULATION_OF_INTERLEUKIN_10_PRODUCTION	16	3	2	4.00E-05	3.19E-02	IL12B;IL23A;IL4
REGULATION_OF_CD4_POSITIVE__ALPHA_BETA_T_CELL_DIFFER ENTIATION	28	4	2	4.00E-05	3.19E-02	IL12B;IL23A;IL23R;IL4
REGULATION_OF_CD4_POSITIVE__ALPHA_BETA_T_CELL_ACTIVA TION	30	4	2	4.00E-05	3.19E-02	IL12B;IL23A;IL23R;IL4
REGULATION_OF_T_CELL_DIFFERENTIATION	82	6	3	6.00E-05	4.53E-02	BAD;IL12B;IL23A;IL23R;IL4;STAT5A
POSITIVE_REGULATION_OF_T_CELL_DIFFERENTIATION	54	6	4	6.00E-05	4.53E-02	BAD;IL12B;IL23A;IL23R;IL4;STAT5A
HAIR_FOLLICLE_MORPHOGENESIS	27	4	2	6.00E-05	4.53E-02	RUNX1;RUNX3;SNAI1;TP63
REGULATION_OF_OSTEOCLAST_DIFFERENTIATION	41	5	3	6.00E-05	4.53E-02	ESRRA;IL12B;IL23A;IL23R;IL4
REGULATION_OF_I_KAPPAB_KINASE_NF_KAPPAB_CASCADE	194	11	2	6.00E-05	4.53E-02	CHUK;FASLG;IKBKE;IL12B;IL23A;NFKBIA;REL;SLC4 4A2;TNFAIP3;TNIP1;TRAF3IP2
REGULATION_OF_TRANSCRIPTION_FACTOR_IMPORT_INTO_NUC LEUS	67	4	2	8.00E-05	5.52E-02	DDX58;IL12B;IL23A;NFKBIA
NEGATIVE_REGULATION_OF_TYPE_I_INTERFERON_PRODUCTIO N	34	4	1	8.00E-05	5.52E-02	DDX58;IFIH1;IKBKE;TNFAIP3
POSITIVE_REGULATION_OF_LEUKOCYTE_DIFFERENTIATION	97	7	3	8.00E-05	5.52E-02	BAD;IL12B;IL23A;IL23R;IL4;RUNX1;STAT5A
POSITIVE_REGULATION_OF_T_CELL_PROLIFERATION	69	5	3	8.00E-05	5.52E-02	IL12B;IL23A;IL23R;IL4;STAT5A
POSITIVE_REGULATION_OF_MYELOID_LEUKOCYTE_DIFFERENTI ATION	37	5	3	8.00E-05	5.52E-02	IL12B;IL23A;IL23R;RUNX1;STAT5A
REGULATION_OF_INTERLEUKIN_12_PRODUCTION	44	4	2	8.00E-05	5.52E-02	IL12B;IL23A;IL23R;REL
HAIR_CYCLE_PROCESS	74	4	1	1.00E-04	6.69E-02	RUNX1;RUNX3;SNAI1;TP63
MOLTING_CYCLE	77	4	1	1.00E-04	6.69E-02	RUNX1;RUNX3;SNAI1;TP63
HAIR_FOLLICLE_DEVELOPMENT	74	4	1	1.00E-04	6.69E-02	RUNX1;RUNX3;SNAI1;TP63
MOLTING_CYCLE_PROCESS	74	4	1	1.00E-04	6.69E-02	RUNX1;RUNX3;SNAI1;TP63

HAIR_CYCLE	77	4	1	1.00E-04	6.69E-02	RUNX1;RUNX3;SNAI1;TP63
POSITIVE_REGULATION_OF_TISSUE_REMODELING	21	2	1	1.20E-04	7.70E-02	IL12B;IL23A
REGULATION_OF_T_CELL_MEDIATED_IMMUNITY	35	3	1	1.40E-04	8.62E-02	IL12B;IL23A;IL23R
POSITIVE_REGULATION_OF_ADAPTIVE_IMMUNE_RESPONSE_BASED_ON_SOMATIC_RECOMBINATION_OF_IMMUNE_RECEPTORS_BUILT_FROM_IMMUNOGLOBULIN_SUPERFAMILY_DOMAINS	45	3	1	1.40E-04	8.62E-02	IL12B;IL23A;IL23R
POSITIVE_REGULATION_OF_T_CELL_MEDIATED_CYTOTOXICITY	14	3	1	1.40E-04	8.62E-02	IL12B;IL23A;IL23R
REGULATION_OF_T_CELL_MEDIATED_CYTOTOXICITY	17	3	1	1.40E-04	8.62E-02	IL12B;IL23A;IL23R
REGULATION_OF_LYMPHOCYTE_DIFFERENTIATION	107	6	3	1.40E-04	8.62E-02	BAD;IL12B;IL23A;IL23R;IL4;STAT5A
POSITIVE_REGULATION_OF_ADAPTIVE_IMMUNE_RESPONSE	48	3	1	1.40E-04	8.62E-02	IL12B;IL23A;IL23R
CHRONIC_MYELOID_LEUKEMIA	73	5	1	1.40E-04	8.62E-02	CHUK;NFKBIA;BAD;STAT5A;RUNX1
POSITIVE_REGULATION_OF_T_CELL_MEDIATED_IMMUNITY	26	3	1	1.40E-04	8.62E-02	IL12B;IL23A;IL23R
ACTIVATION_OF_NF_KAPPAB_IN_B_CELLS	18	3	1	1.60E-04	9.49E-02	CHUK;NFKBIA;REL
REGULATION_OF_ADAPTIVE_IMMUNE_RESPONSE	92	5	2	1.60E-04	9.49E-02	IL12B;IL23A;IL23R;IL4;TNFAIP3
RIG_I_MDA5_MEDIATED_INDUCTION_OF_IFN_ALPHA_BETA_PATHWAYS	44	4	1	1.60E-04	9.49E-02	DDX58;IFIH1;TNFAIP3;IKBKE
REGULATION_OF_ADAPTIVE_IMMUNE_RESPONSE_BASED_ON_SOMATIC_RECOMBINATION_OF_IMMUNE_RECEPTORS_BUILT_FROM_IMMUNOGLOBULIN_SUPERFAMILY_DOMAINS	84	5	2	1.60E-04	9.49E-02	IL12B;IL23A;IL23R;IL4;TNFAIP3

Supplementary Table 7. Enrichment results for regulatory regions among the independent psoriasis signals in different cell types.

Cell types	Enrichment p	Obs	Exp	Obs/Exp
**Th1**	2.23E-22	52	18.74	2.78
**Th0**	1.82E-20	52	19.74	2.63
**CD25-_IL17+_Th17_stim**	1.32E-17	48	19.09	2.51
**CD45RA_CD8**	1.66E-16	43	16.72	2.57
**CD25-_IL17-_Th_stim_MACS**	1.89E-16	49	20.57	2.38
**CD45RO_CD8**	5.72E-16	50	21.64	2.31
**GM12878**	2.27E-15	50	21.84	2.29
**CD25-_CD45RO+_mem**	5.23E-15	44	18.19	2.42
**CD25+_CD127-_Treg**	1.07E-13	44	19.09	2.30
**Th2**	2.39E-13	44	19.04	2.31
**CD25-_CD45RA+_naive**	1.08E-12	43	19.21	2.24
**adult_CD14**	7.70E-12	49	23.88	2.05
**adult_CD20**	4.95E-11	41	18.96	2.16
**B_Cell_Centroblast**	1.59E-09	40	19.36	2.07
**Mobilized_CD34**	3.63E-08	38	19.74	1.92
**colonic_mucosa**	1.98E-07	45	25.42	1.77
**Duodenum_mucosa**	1.12E-06	43	24.96	1.72
**chondrogenic_dif_cells**	1.50E-06	46	27.60	1.67
**K562**	1.78E-06	36	19.97	1.80
**anterior_caudate**	2.58E-06	46	27.97	1.64
**HSMM-myotube**	1.48E-05	46	29.10	1.58
**kidney**	1.55E-05	41	25.06	1.64
**Pancreatic_Islets**	4.51E-05	40	25.25	1.58
**substantia_nigra**	5.08E-05	39	24.39	1.60
**hippocampus_middle**	5.18E-05	42	26.82	1.57
**NH-Osteoblast**	6.92E-05	42	27.11	1.55
**Liver1**	9.07E-05	40	25.77	1.55
**mid_frontal_lobe**	4.47E-04	40	26.94	1.48
**AdiposeNuclei**	4.84E-04	40	27.07	1.48
**inferior_temporal_lobe**	9.85E-04	39	26.87	1.45
**cingulate_gyrus**	2.72E-03	37	26.27	1.41
**angular_gyrus**	4.38E-03	37	26.74	1.38
**HepG2**	1.12E-02	29	20.99	1.38

Supplementary Table 8. Number of markers in strong LD ($r^2 \geq 0.8$) with the most significant marker of each novel locus that overlap with enhancers in different cell/tissue types.

Novel loci (GRCh37)	Nearby genes	Th17	CD8+ (naive)	Th0	Th2
**chr1:78450517**	*FUBP1*	1	0	0	0
**chr1:172675097**	*FASLG*	4	0	1	1
**chr1:206655331**	*IKBKE*	0	0	0	0
**chr10:64369999**	*ZNF365*	1	0	1	0
**chr10:89824771**	*PTEN, KLLN, SNORD74*	1	1	0	0
**chr10:102038641**	*CHUK*	2	2	1	1
**chr11:65593444**	*CFL1, FIBP, FOSL1*	0	0	0	0
**chr12:10597207**	*KLRK1, KLRC4*	0	7	0	0
**chr12:112059557**	*BRAP, MAPKAPK5*	2	0	2	2
**chr12:122668326**	*IL31*	0	0	1	7
**chr13:99950260**	*UBAC2, RN7SKP9*	19	17	14	18
**chr14:98668778:D**	*RP11-61O1.1*	6	5	7	7
**chr15:31637666**	*KLF13*	4	4	4	4
**chr17:73890363**	*TRIM47, TRIM65*	11	7	10	6
**chr18:12857002**	*PTPN2*	0	0	1	1
**chr19:49206417**	*FUT2*	0	0	0	0

Novel loci (GRCh37)	Nearby genes	HSMM	Inferior temporal lobe	K562	Kidney	Liver	Mid-frontal lobe	NH osteoblast	Pancreatic Islets	Substantia nigra
**chr1:78450517**	*FUBP1*	1	1	0	1	0	0	1	1	0
**chr1:172675097**	*FASLG*	0	0	0	0	0	0	0	0	0
**chr1:206655331**	*IKBKE*	0	0	0	0	0	0	0	0	0
**chr10:64369999**	*ZNF365*	3	0	0	0	0	0	2	1	1
**chr10:89824771**	*PTEN, KLLN, SNORD74*	0	1	0	1	1	1	0	1	1
**chr10:102038641**	*CHUK*	2	1	1	2	1	1	1	4	1
**chr11:65593444**	*CFL1, FIBP, FOSL1*	0	0	0	0	0	0	0	0	0
**chr12:10597207**	*KLRK1, KLRC4*	0	3	0	0	0	0	0	0	0
**chr12:112059557**	*BRAP, MAPKAPK5*	3	5	5	4	3	5	4	7	5
**chr12:122668326**	*IL31*	3	0	0	0	0	0	3	0	1
**chr13:99950260**	*UBAC2, RN7SKP9*	5	2	5	8	11	4	9	7	7
**chr14:98668778:D**	*RP11-61O1.1*	0	0	0	0	0	0	0	0	0
**chr15:31637666**	*KLF13*	2	4	1	4	4	4	0	4	4
**chr17:73890363**	*TRIM47, TRIM65*	6	11	5	9	9	11	6	11	11
**chr18:12857002**	*PTPN2*	0	0	0	0	0	0	0	0	0
**chr19:49206417**	*FUT2*	0	0	0	0	0	0	0	0	0

Novel loci (GRCh37)	Nearby genes	Adipose	Angular gyrus	Anterior caudate	Chondrogenic diff	Cingulate gyrus	Colonic mucosa	Duodenum mucosa	HepG2	Hippocampus middle
**chr1:78450517**	*FUBP1*	0	0	1	1	1	0	0	1	1
**chr1:172675097**	*FASLG*	0	0	0	0	0	0	0	0	0
**chr1:206655331**	*IKBKE*	0	0	0	0	0	0	0	0	0
**chr10:64369999**	*ZNF365*	0	0	0	0	0	0	0	0	1
**chr10:89824771**	*PTEN, KLLN, SNORD74*	1	1	1	0	1	1	1	0	1
**chr10:102038641**	*CHUK*	3	1	1	1	1	2	1	2	1
**chr11:65593444**	*CFL1, FIBP, FOSL1*	0	0	0	0	0	0	0	0	0
**chr12:10597207**	*KLRK1, KLRC4*	0	0	3	5	0	0	0	1	0
**chr12:112059557**	*BRAP, MAPKAPK5*	3	5	6	3	5	4	2	1	4
**chr12:122668326**	*IL31*	0	0	0	3	0	0	0	0	1
**chr13:99950260**	*UBAC2, RN7SKP9*	9	2	9	3	6	13	10	2	10
**chr14:98668778:D**	*RP11-61O1.1*	0	0	1	0	0	0	0	0	0
**chr15:31637666**	*KLF13*	4	4	4	0	4	4	4	0	4
**chr17:73890363**	*TRIM47, TRIM65*	10	11	11	10	11	10	9	10	12
**chr18:12857002**	*PTPN2*	0	0	1	0	0	0	0	0	0
**chr19:49206417**	*FUT2*	0	0	0	0	0	0	0	0	0

Supplementary Table 9. Drugs targeting genes from the susceptibility loci

Locus	Gene Target	DrugBank	PharmGKB
**Novel Loci**			
chr1:78450517	*FUBP1*	MGI-114	NA
chr10:89824771	*PTEN*	NA	carboplatin; cisplatin
chr10:102038641	*CHUK*	Aminosalicylic Acid; Mesalazine; Acetylcysteine; Sulfasalazine	NA
chr11:65593444	*CFL1*	Lauryl Dimethylamine-N-Oxide	NA
chr17:73890363	*ACOX1*	(3R)-3-HYDROXYDODECANOIC ACID; Flavin adenine dinucleotide	NA
chr19:49206417	*BCAT2*	Pyridoxal Phosphate; N-[O-Phosphono-Pyridoxyl]-Isoleucine; Pyridoxamine-5'-Phosphate; Alpha-ketoisovalerate; L-Leucine; L-Glutamic Acid; L-Isoleucine	NA
chr19:49206417	*CA11*	Zonisamide	NA
**known loci**			
chr1:67726104	*IL23R*	NA	celecoxib
chr2:163260691	*KCNH7*	Terazosin; Ibutilide; Miconazole; Doxazosin; Prazosin	NA
chr2:163260691	*GCA*	Guanosine-5'-Diphosphate	NA
chr5:96119273	*CAST*	Calcium	NA
chr5:131996445	*IL4*	NA	aspirin
chr5:150467189	*GPX3*	Glutathione	NA
chr5:158829527	*IL12B*	5-Mercapto-2-Nitro-Benzoic Acid; CNTO 1275; ABT-874; humanized SMART Anti-IL-12 Antibody	NA
chr6:31266090	*HLA-C*	NA	ustekinumab; lamotrigine; methazolamide; phenytoin
chr6:31266090	*HLA-B*	N-Formylmethionine	trichloroethylene; interferons; flucloxacillin; acetazolamide; clozapine; dapsone; methazolamide; ribavirin
chr6:111913262	*FYN*	Dasatinib; 1-Methoxy-2-[2-(2-Methoxy-Ethoxy]-Ethane	NA
chr6:138197824	*TNFAIP3*	NA	methotrexate
chr9:32523737	*NDUFB6*	NADH	NA
chr10:75599127	*CAMK2G*	1-tert-butyl-3-(3-methylbenzyl)-1H-pyrazolo[3,4-d]pyrimidin-4-amine; Bosutinib	NA

chr10:75599127	*PLAU*	6-[(Z)-Amino(Imino)Methyl]-N-[4-(Aminomethyl)Phenyl]-4-(Pyrimidin-2-Ylamino)-2-Naphthamide; 6-[N-(1-Isopropyl-3,4-Dihydro-7-Isoquinolinyl)Carbamyl]-2-Naphthalenecarboxamidine; 6-[N-(4-(Aminomethyl)Phenyl)Carbamyl]-2-Naphthalenecarboxamidine; Trans-6-(2-Phenylcyclopropyl)-Naphthalene-2-Carboxamidine; 6-[N-(1-Isopropyl-1,2,3,4-Tetrahydro-7-Isoquinolinyl)Carbamyl]-2-Naphthalenecarboxamidine; 6-Chloro-2-(2-Hydroxy-Biphenyl-3-Yl)-1h-Indole-5-Carboxamidine; 1-[4-(2-oxo-2-phenylethyl)phenyl]guanidine; (2R)-1-(2,6-dimethylphenoxy)propan-2-amine; 6-[N-(4-Ethyl-1,2,3,4-Tetrahydro-6-Isoquinolinyl)Carbamyl]-2-Naphthalenecarboxamidine; CRA_8696; 8-(Pyrimidin-2-Ylamino)Naphthalene-2-Carboximidamide; Plasmin; 4-(2-aminoethoxy)-N-(3-chloro-5-piperidin-1-ylphenyl)-3,5-dimethylbenzamide; 4-(2-aminoethoxy)-N-(2,5-diethoxyphenyl)-3,5-dimethylbenzamide; 4-(2-aminoethoxy)-N-(3-chloro-2-ethoxy-5-piperidin-1-ylphenyl)-3,5-dimethylbenzamide; 2-(2-HYDROXY-BIPHENYL)-1H-BENZOIMIDAZOLE-5-CARBOXAMIDINE; 6-FLUORO-2-(2-HYDROXY-3-ISOBUTOXY-PHENYL)-1H-BENZOIMIDAZOLE-5-CARBOXAMIDINE; 6-FLUORO-2-[2-HYDROXY-3-(2-METHYL-CYCLOHEXYLOXY)-PHENYL]-1H-INDOLE-5-CARBOXAMIDINE; N-(4-CARBAMIMIDOYL-3-CHORO-PHENYL)-2-HYDROXY-3-IODO-5-METHYL-BENZAMIDE; 6-[(Z)-AMINO(IMINO)METHYL]-N-[3-(CYCLOPENTYLOXY)PHENYL]-2-NAPHTHAMIDE; Urokinase; 2-(2-Hydroxy-5-Methoxy-Phenyl)-1h-Benzoimidazole-5-Carboxamidine; Thieno[2,3-B]Pyridine-2-Carboxamidine; Benzamidine; CRA_10655; [2,4,6-Triisopropyl-Phenylsulfonyl-L-[3-Amidino-Phenylalanine]]-Piperazine-N'-Beta-Alanine; 4-Iodobenzo[B]Thiophene-2-Carboxamidine; 7-Methoxy-8-[1-(Methylsulfonyl)-1h-Pyrazol-4-Yl]Naphthalene-2-Carboximidamide; 4-(2-AMINOETHOXY)-3,5-DICHLORO-N-[3-(1-METHYLETHOXY)PHENYL]BENZAMIDE; 2-Amino-5-Hydroxy-Benzimidazole; Amiloride; 2-(2-Hydroxy-Phenyl)-1h-Benzoimidazole-5-Carboxamidine; N-(1-Adamantyl)-N'-(4-Guanidinobenzyl)Urea; 6-(N-Phenylcarbamyl)-2-Naphthalenecarboxamidine	NA
chr10:81032532	*PPIF*	L-Proline; Cyclosporine; 7-AMINO-4-METHYL-CHROMEN-2-ONE; 1-Methoxy-2-[2-(2-Methoxy-Ethoxy]-Ethane	NA
chr11:64135298	*ESRRA*	Troglitazone; 1-CYCLOHEXYL-N-{[1-(4-METHYLPHENYL)-1H-INDOL-3-YL]METHYL}METHANAMINE	NA
chr11:64135298	*PRDX5*	Diminazene; Auranofin; Benzoic Acid	NA
chr11:64135298	*RPS6KA4*	Riboflavin Monophosphate	NA
chr11:64135298	*BAD*	ABT-263	NA
chr12:56750204	*IL23A*	ABT-874	NA
chr12:56750204	*CS*	Nitromethyldethia Coenzyme A; Oxaloacetate Ion; Trifluoroacetonyl Coenzyme A; Alpha-Fluoro-Carboxymethyldethia Coenzyme a Complex; Malate Ion; Coenzyme A; Citric Acid	NA
chr14:35832666	*NFKBIA*	Acetylsalicylic acid	NA
chr14:35832666	*PSMA6*	(3AR,6R,6AS)-6-((S)-((S)-CYCLOHEX-2-ENYL)(HYDROXY)METHYL)-6A-METHYL-4-OXO-HEXAHYDRO-2H-FURO[3,2-C]PYRROLE-6-CARBALDEHYDE; N-[(1R)-1-(DIHYDROXYBORYL)-3-METHYLBUTYL]-N-(PYRAZIN-2-YLCARBONYL)-L-PHENYLALANINAMIDE	NA
chr16:31004812	*PRSS53*	NA	warfarin; phenprocoumon; fluindione

chr16:31004812	*VKORC1*	Warfarin; Menadione; Acenocoumarol; Dicoumarol; Phenindione; Phenprocoumon	fluindione; coumarin
chr16:31004812	*STX4*	NA	warfarin; phenprocoumon
chr17:26124908	*NOS2*	Aminoguanidine; S-Ethylisothiourea; (3R)-3-[(1,2,3,4-tetrahydroisoquinolin-7-yloxy)methyl]-2,3-dihydrothieno[2,3-f][1,4]oxazepin-5-amine; 1-(6-CYANO-3-PYRIDYLCARBONYL)-5',8'-DIFLUOROSPIRO[PIPERIDINE-4,2'(1'H)-QUINAZOLINE]-4'-AMINE; KD7040; N,N-dimethylarginine; L-Citrulline; 7-Nitroindazole; 4r-Fluoro-N6-Ethanimidoyl-L-Lysine; N-[2-(4-AMINO-5,8-DIFLUORO-1,2-DIHYDROQUINAZOLIN-2-YL)ETHYL]-3-FURAMIDE; ACCLAIM; Ethylisothiourea; N-(3-(Aminomethyl)Benzyl)Acetamidine; 4-(1,3-BENZODIOXOL-5-YLOXY)-2-[4-(1H-IMIDAZOL-1-YL)PHENOXY]-6-METHYLPYRIMIDINE; (3S)-1-(1,3-BENZODIOXOL-5-YLMETHYL)-3-[4-(1H-IMIDAZOL-1-YL)PHENOXY]PIPERIDINE; 7,8-Dihydro-L-Biopterin; 5-(4'-AMINO-1'-ETHYL-5',8'-DIFLUORO-1'H-SPIRO[PIPERIDINE-4,2'-QUINAZOLINE]-1-YLCARBONYL)PICOLINONITRILE; 4-(1H-IMIDAZOL-1-YL)PHENOL; Dexamethasone; N-[2-(1,3-BENZODIOXOL-5-YL)ETHYL]-1-[2-(1H-IMIDAZOL-1-YL)-6-METHYLPYRIMIDIN-4-YL]-D-PROLINAMIDE; N-[2-(6-AMINO-4-METHYLPYRIDIN-2-YL)ETHYL]-4-CYANOBENZAMIDE; ETHYL 4-[(4-METHYLPYRIDIN-2-YL)AMINO]PIPERIDINE-1-CARBOXYLATE; L-Thiocitrulline; Thiocoumarin; 1-[4-(AMINOMETHYL)BENZOYL]-5'-FLUORO-1'H-SPIRO[PIPERIDINE-4,2'-QUINAZOLIN]-4'-AMINE; (6r,1'r,2's)-5,6,7,8 Tetrahydrobiopterin; N-(4-(2-((3-Chlorophenylmethyl)Amino)Ethyl)Phenyl)-2-Thiophecarboxamidine; 6-Nitroindazole; L-Arginine; N-Omega-Hydroxy-L-Arginine; ETHYL 4-[(4-CHLOROPYRIDIN-2-YL)AMINO]PIPERIDINE-1-CARBOXYLATE; 7,8-Dihydrobiopterin; 5-Nitroindazole; Miconazole; N-Omega-Propyl-L-Arginine; 4-({4-[(4-methoxypyridin-2-yl)amino]piperidin-1-yl}carbonyl)benzonitrile; (2S)-2-methyl-2,3-dihydrothieno[2,3-f][1,4]oxazepin-5-amine; 4-(1,3-BENZODIOXOL-5-YLOXY)-2-[4-(1H-IMIDAZOL-1-YL)PHENOXY]PYRIMIDINE; Triflusal; 3-Bromo-7-Nitroindazole	isoniazid; rifampin; doxorubicin
chr17:40561579	*STAT3*	NA	interferons
chr17:78178893	*GAA*	Acarbose; AT2220; Miglitol	NA
chr19:10463118	*TYK2*	3-{(3R,4R)-4-methyl-3-[methyl(7H-pyrrolo[2,3-d]pyrimidin-4-yl)amino]piperidin-1-yl}-3-oxopropanenitrile; 2-(1,1-DIMETHYLETHYL)9-FLUORO-3,6-DIHYDRO-7H-BENZ[H]-IMIDAZ[4,5-F]ISOQUINOLIN-7-ONE	NA
chr19:10463118	*ICAM1*	Hyaluronan; Natalizumab	NA
chr19:10463118	*PDE4A*	PDE4; Theophylline; AN2728; Dyphylline; Roflumilast; 4-[8-(3-nitrophenyl)-1,7-naphthyridin-6-yl]benzoic acid; Iloprost; Enprofylline; Drotaverine; Pentoxifylline; Oxtriphylline; Piclamilast; Ibudilast; (4R)-4-(3-butoxy-4-methoxybenzyl)imidazolidin-2-one; Tofisopam; Ketotifen; Dipyridamole	NA

Supplementary Note

Annotated functions of notable candidate genes within newly identified psoriasis susceptibility loci

***FUBP1* (1p31.1):** *FUBP1* encodes a single stranded DNA-binding protein that modulates c-Myc mRNA levels. As a transcription factor c-Myc controls the expression of about 10% of cellular genes, including those essential for cell proliferation, differentiation and apoptosis [5-8]. FUBP1 also directly represses the cell cycle inhibitor p21 [9], which also plays an important role in myeloid cell differentiation with loss of p21 inhibiting both DC and macrophage differentiation [10].

FASLG* (1q24.3)*: *FASLG* encodes Fas ligand (CD95), a type-II transmembrane protein that belongs to the tumor necrosis factor (TNF) family. Fas ligand/receptor interactions play an important role in the regulation of the immune system [11]. Although apoptosis is the most studied outcome of Fas:Fas ligand binding [11], Fas receptor engagement includes promotion of inflammatory responses in cell such as macrophages [12], dendritic cells [13,14], and keratinocytes [15]. The Fas:Fas ligand system may interact with NF-κB via A20 (*TNFAIP3*) [16], a known risk gene for psoriasis [1].

IKBKE* (1q32.1)*: IKBKE encodes IKKε, which belongs to the IκB kinase family, and is essential for regulation of antiviral signaling pathways. IKKε is activated downstream of cytosolic RNA/DNA sensors, and mediates phosphorylation and activation of transcription factors, including IRF3 and IRF7, and NF-κB [17-19].

ZNF365 (10q21.2): *ZNF365* encodes a zinc-finger binding protein. It is induced by DNA double-strand breakage and is involved in the homologous recombination pathway and maintains genome integrity during DNA replication. It has been reported to be a susceptibility locus for Crohn's disease [20], and atopic dermatitis [21].

PTEN, KLLN, (10q23.31): *PTEN* is a tumor suppressor that inhibits cell proliferation through inactivation of the PI3 kinase/Akt pathway. Keratinocyte-specific Pten deficiency in mice results in epidermal hyperplasia and hyperkeratosis [22]. PTEN has been reported to be suppressed in psoriatic skin compared to normal skin [23]. *KLLN* is a high-affinity DNA-binding protein, downstream of p53 and necessary for p53-induced apoptosis [24].

***CHUK* (10q24.31):** CHUK encodes IKK1/IKKα. It is one of three core proteins of the IKK complex and has a critical role in regulating NF-κB activity. It has been reported to have an important role in inflammatory responses [25,26] and epidermal differentiation [27].

***CFL1, FIBP, FOSL1* (11q13.1):** *CFL1* encodes for cofilin, which is a widely distributed intracellular actin-modulating protein and may have a role in regulating dendritic and T-cell migration and T-cell activation [28,29]. *FIBP* encodes for acidic fibroblast growth factor (aFGF) intracellular binding protein. It is mainly found in the nucleus and thought to be involved in the intracellular function of aFGF [30]. *FOSL1* is a member of the AP-1 transcription factor and has been shown to have a role in endothelial assembly into capillary tubes [31].

***KLRK1, KLRC4* (12p13.2):** *KLRK1* encodes NKG2D, a killer cell lectin-like receptor. It is an activating receptor that triggers natural killer (NK) cell CD8+ T-cells, but is normally absent from CD4+ T-cells. NKG2D is also present on most gamma-delta T-cells. Its ligands are stress induced proteins such as MICA [32]. *KLRC4* encodes NKG2F. It is expressed intracellularly in NK cells but is unable to bind with CD94 and has been thought to be a vestigial gene product [33].

BRAP, MAPKAPK5 (12q24.12): *BRAP* may function as a cytoplasmic retention protein and play a role in the cytoplasmic translocation of p21 [34],. *MAPKAP5* encodes a protein kinase regulated by p38. It is activated in response to cellular stress and pro-inflammatory cytokines [35] and is involved in tumor suppression, cell motility and cell cycle regulation [36].

***IL31* (12q24.31):** *IL31* encodes IL-31, which is a member of the IL-6 cytokine family. It is thought to be mainly a Th2– derived cytokine that has been implicated in the pathogenesis of atopic dermatitis [37] and promotes Th2-driven inflammation [38].

***UBAC2* (13q32.3):** UBAC2 has an ubiquitin-associated domain and is predicted to be involved in ubiquitination processes [39]. Polymorphisms in this gene have been associated with Bechet's disease [39] and cutaneous basal and squamous cell carcinoma [40]. The function of the protein encoded by UBAC2 is not known.

***RP11-61O1.1* (14q32.2):** *RP11-61O1.1* encodes a novel lincRNA., whose function is currently unknown.

***KLF13* (15q13.3)**: *KLF13* encodes for Kruppel-like factor 13, and belongs to a family of transcription factors that have C2H2 motif. KLF13 is the dominant regulator of the chemokine RANTES (CCL5) in T-cells [41] and has a role in regulating IL-4 expression in CD4+T-cells [42].

***TRIM47, TRIM65* (17q25.1)**: *TRIM47* and *TRIM65* encode tripartite motif-containing protein 47 and 65.. Over 60 TRIM proteins are known and many of these are induced by type I and type II interferons and play a crucial role in pathogen-recognition and anti-viral responses [43]. Members of the TRIM family interact with RIG1/DDX58 a previously implicated risk gene for psoriasis [4].

***PTPN2* (18p11.21):** *PTPN2* encodes protein-tyrosine phosphatase, T-cell. PTPN2 has various roles in T-cell biology. Loss of *PTPN2* in the T-cell compartment leads to enhanced induction of Th1 and Th17 cells along with impaired induction of regulatory T-cells [44]. *PTPN2* is a critical mediator of peripheral tolerance by restraining CD8+ T-cell responses after antigen-cross-presentation [45].

***FUT2* (19q13.33):** *FUT2* encodes alpha – (1,2) fucosyltransferase with has a role in glycosylation of proteins. It regulates expression of the Lewis ABO blood group antigens on the surface of epithelial cells [46] and determines the secretion status of the ABO antigens. Risk variants in *FUT2* have been associated with Crohn's disease [47,48].

Supplementary References

1. Nair, R.P. *et al.* Genome-wide scan reveals association of psoriasis with IL-23 and NF-kappaB pathways. *Nat Genet* **41**, 199-204 (2009).
2. Ellinghaus, E. *et al.* Genome-wide association study identifies a psoriasis susceptibility locus at TRAF3IP2. *Nat Genet* **42**, 991-5 (2010).
3. Strange, A. *et al.* A genome-wide association study identifies new psoriasis susceptibility loci and an interaction between HLA-C and ERAP1. *Nat Genet* **42**, 985-90 (2010).
4. Tsoi, L.C. *et al.* Identification of 15 new psoriasis susceptibility loci highlights the role of innate immunity. *Nat Genet* **44**, 1341-8 (2012).
5. de Nigris, F. *et al.* c-Myc oncoprotein: cell cycle-related events and new therapeutic challenges in cancer and cardiovascular diseases. *Cell Cycle* **2**, 325-8 (2003).
6. Hoffman, B. & Liebermann, D.A. Apoptotic signaling by c-MYC. *Oncogene* **27**, 6462-72 (2008).
7. Levens, D. Disentangling the MYC web. *Proc Natl Acad Sci U S A* **99**, 5757-9 (2002).
8. Pelengaris, S., Khan, M. & Evan, G. c-MYC: more than just a matter of life and death. *Nat Rev Cancer* **2**, 764-76 (2002).
9. Rabenhorst, U. *et al.* Overexpression of the far upstream element binding protein 1 in hepatocellular carcinoma is required for tumor growth. *Hepatology* **50**, 1121-9 (2009).
10. Kramer, J.L., Baltathakis, I., Alcantara, O.S. & Boldt, D.H. Differentiation of functional dendritic cells and macrophages from human peripheral blood monocyte precursors is dependent on expression of p21 (WAF1/CIP1) and requires iron. *Br J Haematol* **117**, 727-34 (2002).
11. Cullen, S.P. & Martin, S.J. Fas and TRAIL 'death receptors' as initiators of inflammation: Implications for cancer. *Semin Cell Dev Biol* (2015).
12. Park, D.R. *et al.* Fas (CD95) induces proinflammatory cytokine responses by human monocytes and monocyte-derived macrophages. *J Immunol* **170**, 6209-16 (2003).
13. Guo, Z., Zhang, M., Tang, H. & Cao, X. Fas signal links innate and adaptive immunity by promoting dendritic-cell secretion of CC and CXC chemokines. *Blood* **106**, 2033-41 (2005).
14. Rescigno, M. *et al.* Fas engagement induces the maturation of dendritic cells (DCs), the release of interleukin (IL)-1beta, and the production of interferon gamma in the absence of IL-12 during DC-T cell cognate interaction: a new role for Fas ligand in inflammatory responses. *J Exp Med* **192**, 1661-8 (2000).
15. Farley, S.M. *et al.* Fas ligand elicits a caspase-independent proinflammatory response in human keratinocytes: implications for dermatitis. *J Invest Dermatol* **126**, 2438-51 (2006).
16. Malewicz, M., Zeller, N., Yilmaz, Z.B. & Weih, F. NF kappa B controls the balance between Fas and tumor necrosis factor cell death pathways during T cell receptor-induced apoptosis via the expression of its target gene A20. *J Biol Chem* **278**, 32825-33 (2003).
17. Sharma, S. *et al.* Triggering the interferon antiviral response through an IKK-related pathway. *Science* **300**, 1148-51 (2003).
18. Fitzgerald, K.A. *et al.* IKKepsilon and TBK1 are essential components of the IRF3 signaling pathway. *Nat Immunol* **4**, 491-6 (2003).
19. Mattioli, I. *et al.* Inducible phosphorylation of NF-kappa B p65 at serine 468 by T cell costimulation is mediated by IKK epsilon. *J Biol Chem* **281**, 6175-83 (2006).
20. Yang, S.K. *et al.* Immunochip analysis identification of 6 additional susceptibility loci for Crohn's disease in Koreans. *Inflamm Bowel Dis* **21**, 1-7 (2015).
21. Hirota, T. *et al.* Genome-wide association study identifies eight new susceptibility loci for atopic dermatitis in the Japanese population. *Nat Genet* **44**, 1222-6 (2012).

22. Suzuki, A. *et al.* Keratinocyte-specific Pten deficiency results in epidermal hyperplasia, accelerated hair follicle morphogenesis and tumor formation. *Cancer Res* **63**, 674-81 (2003).
23. Li, Y. *et al.* Downregulation of PTEN expression in psoriatic lesions. *Int J Dermatol* **53**, 855-60 (2014).
24. Cho, Y.J. & Liang, P. Killin is a p53-regulated nuclear inhibitor of DNA synthesis. *Proc Natl Acad Sci U S A* **105**, 5396-401 (2008).
25. Lawrence, T., Bebien, M., Liu, G.Y., Nizet, V. & Karin, M. IKKalpha limits macrophage NF-kappaB activation and contributes to the resolution of inflammation. *Nature* **434**, 1138-43 (2005).
26. Li, X. *et al.* IKKalpha, IKKbeta, and NEMO/IKKgamma are each required for the NF-kappa B-mediated inflammatory response program. *J Biol Chem* **277**, 45129-40 (2002).
27. Li, Q. *et al.* IKK1-deficient mice exhibit abnormal development of skin and skeleton. *Genes Dev* **13**, 1322-8 (1999).
28. Xu, Y. *et al.* Dendritic cell motility and T cell activation requires regulation of Rho-cofilin signaling by the Rho-GTPase activating protein myosin IXb. *J Immunol* **192**, 3559-68 (2014).
29. Samstag, Y., John, I. & Wabnitz, G.H. Cofilin: a redox sensitive mediator of actin dynamics during T-cell activation and migration. *Immunol Rev* **256**, 30-47 (2013).
30. Kolpakova, E., Frengen, E., Stokke, T. & Olsnes, S. Organization, chromosomal localization and promoter analysis of the gene encoding human acidic fibroblast growth factor intracellular binding protein. *Biochem J* **352 Pt 3**, 629-35 (2000).
31. Evellin, S. *et al.* FOSL1 controls the assembly of endothelial cells into capillary tubes by direct repression of alphav and beta3 integrin transcription. *Mol Cell Biol* **33**, 1198-209 (2013).
32. Zou, Y. & Stastny, P. Role of MICA in the immune response to transplants. *Tissue Antigens* **76**, 171-6 (2010).
33. Kim, D.K. *et al.* Human NKG2F is expressed and can associate with DAP12. *Mol Immunol* **41**, 53-62 (2004).
34. Asada, M. *et al.* Brap2 functions as a cytoplasmic retention protein for p21 during monocyte differentiation. *Mol Cell Biol* **24**, 8236-43 (2004).
35. New, L. *et al.* PRAK, a novel protein kinase regulated by the p38 MAP kinase. *EMBO J* **17**, 3372-84 (1998).
36. Lindin, I., Wuxiuer, Y., Ravna, A.W., Moens, U. & Sylte, I. Comparative molecular dynamics simulations of mitogen-activated protein kinase-activated protein kinase 5. *Int J Mol Sci* **15**, 4878-902 (2014).
37. Sonkoly, E. *et al.* IL-31: a new link between T cells and pruritus in atopic skin inflammation. *J Allergy Clin Immunol* **117**, 411-7 (2006).
38. Stott, B. *et al.* Human IL-31 is induced by IL-4 and promotes TH2-driven inflammation. *J Allergy Clin Immunol* **132**, 446-54 e5 (2013).
39. Sawalha, A.H. *et al.* A putative functional variant within the UBAC2 gene is associated with increased risk of Behcet's disease. *Arthritis Rheum* **63**, 3607-12 (2011).
40. Nan, H. *et al.* Genome-wide association study identifies novel alleles associated with risk of cutaneous basal cell carcinoma and squamous cell carcinoma. *Hum Mol Genet* **20**, 3718-24 (2011).
41. Song, A., Chen, Y.F., Thamatrakoln, K., Storm, T.A. & Krensky, A.M. RFLAT-1: a new zinc finger transcription factor that activates RANTES gene expression in T lymphocytes. *Immunity* **10**, 93-103 (1999).
42. Kwon, S.J. *et al.* KLF13 cooperates with c-Maf to regulate IL-4 expression in CD4+ T cells. *J Immunol* **192**, 5703-9 (2014).
43. Ozato, K., Shin, D.M., Chang, T.H. & Morse, H.C., 3rd. TRIM family proteins and their emerging roles in innate immunity. *Nat Rev Immunol* **8**, 849-60 (2008).

44. Spalinger, M.R. *et al.* PTPN2 controls differentiation of CD4 T cells and limits intestinal inflammation and intestinal dysbiosis. *Mucosal Immunol* (2014).
45. Wiede, F., Ziegler, A., Zehn, D. & Tiganis, T. PTPN2 restrains CD8(+) T cell responses after antigen cross-presentation for the maintenance of peripheral tolerance in mice. *J Autoimmun* **53**, 105-14 (2014).
46. Koda, Y., Soejima, M., Wang, B. & Kimura, H. Structure and expression of the gene encoding secretor-type galactoside 2-alpha-L-fucosyltransferase (FUT2). *Eur J Biochem* **246**, 750-5 (1997).
47. McGovern, D.P. *et al.* Fucosyltransferase 2 (FUT2) non-secretor status is associated with Crohn's disease. *Hum Mol Genet* **19**, 3468-76 (2010).
48. Franke, A. *et al.* Genome-wide meta-analysis increases to 71 the number of confirmed Crohn's disease susceptibility loci. *Nat Genet* **42**, 1118-25 (2010).
